# Multilevel trait responses of liana *Hedera helix* L. to environmental gradients in urban forest ecosystems

**DOI:** 10.1038/s41598-025-23815-0

**Published:** 2025-11-17

**Authors:** Olena Blinkova, Roma Żytkowiak, Justyna Wiland-Szymańska, Mateusz Sowelo, Małgorzata Jaźwa, Ewelina Ratajczak, Andrzej M. Jagodziński

**Affiliations:** 1 Institute of Dendrology, Polish Academy of Sciences, Parkowa 5, 62-035 Kórnik, Poland; 2 Taras Shevchenko Lugansk National University, Koval 3, Poltava, 36-003 Ukraine; 3 Botanical Garden of Adam Mickiewicz University, Dąbrowskiego 165, 60-594 Poznan, Poland; 4 Faculty of Biology, Adam Mickiewicz University in Poznań, Uniwersytetu Poznańskiego 6, 61-614 Poznan, Poland; 5 Institute of Biology, University of Opole, Oleska 22, 45-052 Opole, Poland

**Keywords:** *Hedera helix*, Urban forest, Trait integration, Environmental factor, Ecological indicator, Ecology, Ecology, Environmental sciences, Plant sciences

## Abstract

Urban forest ecosystems face increasing abiotic stress due to climate change and anthropogenic pressure, requiring robust indicators of plant adaptation. *Hedera helix* L., a widespread liana with shoot dimorphism, plays a key role in temperate urban woodlands but remains understudied in terms of multilevel responses to environmental gradients. We assessed vegetative and generative shoots with healthy or damaged leaves across heterogeneous urban forest sites to identify functional responses to volumetric water content (VWC), temperature (t), electrical conductivity (ES), and daily light integral (DLI). VWC and DLI emerged as the key drivers of trait variability. The multilevel adaptive trait responses demonstrated the ecological flexibility of a dominant urban liana. We developed a novel Integrative Ecological Index (IEI) based on normalized trait sub-indices, which captures multilevel plant responses to environmental stress and enables quantitative assessment of urban habitat conditions. IEI was highest in healthy vegetative shoots and lowest in damaged generative shoots. Our findings demonstrate the ecological plasticity of *H. helix* and its potential as a sensitive bioindicator. This interdisciplinary study provides a scalable trait-based tool to evaluate adaptive capacity and microhabitat conditions in heterogeneous urban environments.

**Supplementary Information:**

The online version contains supplementary material available at 10.1038/s41598-025-23815-0.

## Introduction

Plant functional traits determine species development and form the basis for explaining spatial patterns of species distribution^1,2^. The spectrum of these traits is closely linked to ecological strategies^3^ and reflects the trade-off between rapid resource acquisition and resource conservation, known as the leaf economics spectrum^4^. Adaptive modifications in plant organ traits may arise from plastic responses within individuals over seasonal cycles or across species along environmental gradients^5^. The ability of plants to exploit available resources in complementary ways further enables the coexistence of multiple strategies in resource-rich environments^4,5^. Increasing attention is being paid to how morphological, physiological, and biochemical traits shape plant responses to fine-scale gradients of light, water, and temperature in forest ecosystems^2,3^. For lianas, such traits underpin their ability to maintain viability in heterogeneous environments, particularly under conditions of microclimatic variability and anthropogenic pressure^6^. Lianas play an important role in the functioning of forest ecosystems and occur in forests across different climate zones of the world, including tropical, subtropical, temperate, and even boreal regions^6^. Lianas contribute to forest structure and dynamics^7–11^. A liana species that is important in Europe is English ivy (*Hedera helix* L.), which often acts as a dominant or subdominant species in the forest understory, forming extensive ground and trunk cover and influencing microclimatic and light conditions near the forest floor.

*H. helix* is a perennial liana from the family *Araliaceae*^12^, often long-lived^13^. This evergreen climber grows in temperate deciduous forests. The shoots are differentiated into juvenile shoots—long and creeping, sometimes hairy—and mature shoots—not developing aerial rootlets and protruding from the supports, hanging over time^14–16^. *H. helix* has a native range spanning the Mediterranean Basin and western, central, and eastern Europe, extending northward to southern Norway, Sweden, and Latvia, and eastward through Turkey and the Caucasus to western Iran^17,18^. Within Poland, it is widely distributed and commonly associated with temperate deciduous and alluvial forests^19,20^. In addition to moist, shady forests, it can also grow well in dry and sunny places and on rocky debris. It is a shade-tolerant species that is tolerant of both slightly acidic and alkaline soils, but it prefers fresh soils rich in humus^16^. Despite its natural presence in temperate forests, *H. helix* is increasingly common in urban green areas, where microclimatic conditions and anthropogenic factors shape its subpopulation structure. The species also has strong adaptability to varying soil moisture and light levels^21^. The plant is tolerant to drought, shade, cold, and frost and is resistant to diseases and insect pests^22–24^. *H. helix* is an indicator of fairly warm growing conditions in both lowland and high mountain areas, especially in submontane to temperate regions^7^. Within its natural range, its occurrence is limited by the isotherm corresponding to a mean January temperature ≥  − 4 °C^25,26^. In colder regions, low temperatures restrict its functional performance, leading to predominantly vegetative reproduction, rare flowering, and limited sexual regeneration^25–27^.

High ecological tolerance and structural plasticity enable *H. helix* to establish across diverse environments; the same traits together with efficient climbing via adventitious roots and clonal spread from nodal rooting^14,24^ and long-distance avian dispersal of berries^14^ underpin its invasive potential. Facilitated by widespread ornamental planting, the species is now naturalized and frequently invasive across multiple regions, including the Americas, Oceania and North Africa^28–31^. In parts of its native range, including Poland, dense ivy cover can form extensive monospecific mats that suppress native understory vegetation and alter local microclimatic and light conditions, creating so-called “ivy deserts”^32–34^ (Fig. S1).

Within its native range, *H. helix* was historically protected in Poland (1946–2014) due to its limited distribution, but this status was revoked following a notable increase in recorded populations^35^. At the northern range margin the species remains regionally endangered^36^. The species also frequently colonizes semi-natural habitats such as old parks, cemeteries, and botanical gardens^37–39^, where distinguishing natural from anthropogenically influenced stands can be challenging. Increasing ivy cover can substantially alter understory species composition and ecosystem dynamics, particularly where these shifts coincide with climate-driven changes and anthropogenic pressures such as deforestation and habitat fragmentation^40–42^.

The influence of *H. helix* on plant communities can be considered in different categories. Based on its ability to survive under different environmental conditions according to Grime’s model, ivy represents the stress tolerance/competitor strategy type^43^. However, in studies of oak woodlands in Samsun, northern Turkey, the results support the C/CSR strategy as climbers, whereby competition was the major pressure factor but disturbance and stress also had effects on disturbed habitats^44^. The competition for light, water and nutrients between lianas and plant hosts influences the dynamics of individual trees and entire forest communities^42^. This process can inhibit the growth of vines and the production of generative organs or increase the mortality of host trees^11,40,45^. In observations and studies conducted in forests in Germany^46^, England^14^ and France^47^, no higher mortality or significant differences were observed between the growth rings of trees overgrown with *H. helix* and trees free from the liana. On the other hand, studies have highlighted the negative impact of ivy on host trees^48^. *H. helix* is susceptible to *Xylella fastidiosa*, a bacterium that causes a number of plant diseases, including in trees, especially in damaged forest habitats, and may play an important role in the spread of this pathogen^49^.

Despite these potential ecological risks, *H. helix* also provides numerous ecosystem services and contributes significantly to biodiversity and urban ecological health. As an intensively branching liana, it is also a source of food and shelter for many animal species^50,51^. Within its natural range, it is an indicator of biodiverse forests^52^ and is an important component of tree-related microhabitats^53^. It is also a species that can capture particulate matter from the air, making it a valuable plant for urban green areas management^54,55^. Thus, ivy can play a significant role in urban environments by improving local air quality and reducing the urban heat island effect^56^. In addition, *H. helix* has medicinal properties, such as anti-inflammatory and antibacterial effects, and plays an important role in traditional medicine^57–59^.

In the context of increasing anthropogenic pressure and environmental heterogeneity in urban ecosystems, bioindication studies that integrate multiple levels of biological organization are essential. Assessing plant responses across individual and population scales using morphological, functional, and biochemical traits enables the identification of adaptive strategies and environmental stress responses^60,61^. Urban forest ecosystems exhibit pronounced microhabitat heterogeneity, where anthropogenic pressures and fine-scale gradients of soil moisture, light, temperature, and electrical conductivity shape the realized ecological niches of plants. Moreover, organ damage acts as an extra stress factor, increasing the variability of morphological, functional, and biochemical traits.

In plant functional ecology, the concept of trait integration describes the coordinated variation of phenotypic traits in response to abiotic and anthropogenic factors. Such covariation reflects an adaptive strategy aimed at optimizing resource use and maintaining functional stability under changing environmental conditions^54^. Functional leaf traits (e.g., SLA, LMA, and hydration indices) provide key insights into resource-use strategies and water balance regulation along fine-scale urban gradients^21^. Physiological markers of the pigment system (Chl a, Chl b, carotenoids, and the Chl a/b ratio) sensitively capture early shifts in photochemical efficiency and light stress^61^. Proteins play pivotal roles in plant adaptation to abiotic stressors such as drought, flooding, and extreme temperatures by regulating metabolism, signal transduction, and transcriptional responses^62^. A comprehensive assessment of these morphological, physiological, and biochemical indicators substantially enhances the diagnostic potential of multilevel bioindication in heterogeneous urban environments^54,61^. This provides a foundation for the quantitative assessment of urban forest ecosystem condition and highlights the potential of key species as model organisms for testing bioindication hypotheses in functional ecology.

Owing to its wide ecological tolerance, shoot dimorphism, and structural plasticity, *H. helix* represents a valuable model species for multilevel bioindication in urban woody habitats. Its high phenotypic plasticity and consistent adaptive responses provide a unique opportunity to explore plant response mechanisms and adaptive strategies under heterogeneous urban conditions. A complex analytical approach provides comprehensive insights into the species’ ecological responses to key abiotic drivers. In *H. helix*, alterations in protein synthesis and regulation may accompany changes in pigment composition and leaf hydration status, representing an additional mechanism of stress adaptation under heterogeneous urban conditions.

However, despite the accumulating evidence on the ecological plasticity of *H. helix*, the interactive effects of urban abiotic gradients on shoot morphology, pigment system stability, and hydration status remain poorly quantified at the level of the shoot type and leaf condition. These unresolved mechanisms provide the rationale for testing how ivy responds to urban environmental gradients across multiple organizational levels, as formulated in the following two hypotheses.

H1) *H. helix* exhibits significant and consistent differences in morphological, functional, and biochemical traits between vegetative and generative shoots, as well as between healthy and damaged leaves, with these differences being shaped by fine-scale urban environmental gradients.

H2) Vegetative shoots show stronger associations with abiotic factors and demonstrate partially coordinated adjustments in morphology, pigmentation, and hydration status, reflecting their integrative role in microhabitat-level acclimation and their context-dependent ecological resilience under heterogeneous urban conditions.

The aim of the present study was to comprehensively assess the morphological, physiological, and biochemical responses of *H. helix* shoots to environmental gradients, including light intensity, the soil volumetric water content, electrical conductivity, and soil temperature. By comparing healthy and damaged leaves on vegetative and generative shoots across contrasting microhabitats, we aimed to examine the specific effects of key abiotic factors on shoot morphology, pigment composition, and the leaf hydration status. This multilevel approach thus provides insights into the mechanisms of liana adaptability and subpopulation dynamics in urban forest ecosystems^63^.

## Materials and methods

**Fig. 1:**
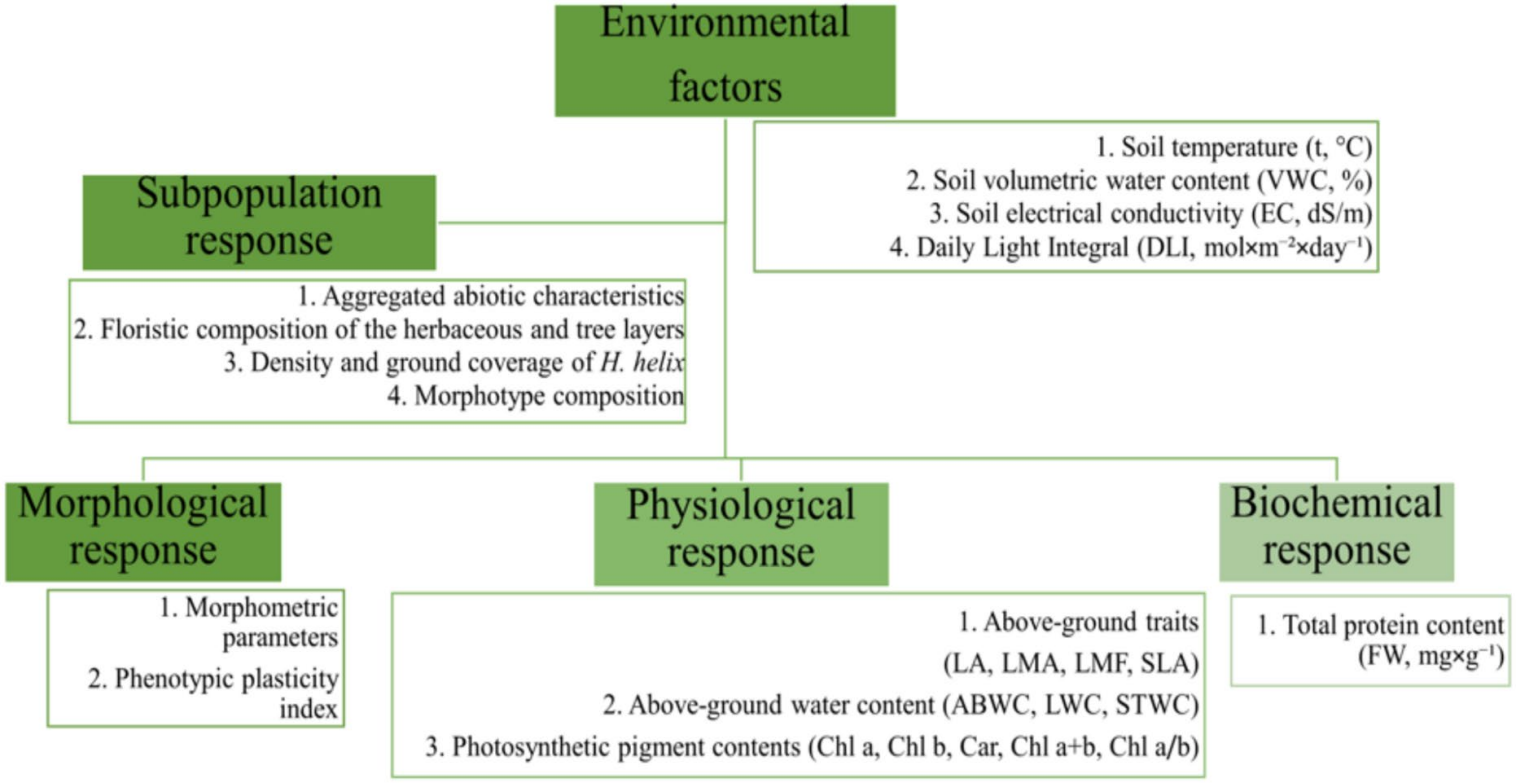
*H. helix* responses across biological scales.

In Poland, *H. helix* occurs throughout the entire area, less frequently in the center, in Podlasie and Lublin, very rarely in the northern part of Mazovia and in the Suwałki region^19^. In nature, it usually occurs in deciduous forests^16^, and it is associated with the zonal temperate broad-leaved forests *Carpino-Fagetea sylvaticae* and alluvial forests and scrub *Alno glutinosae–Populetea albae* communities^20^. To capture its responses, this study employed a multilevel analytical approach to assess the impacts of urban environmental gradients on *H. helix* responses across hierarchical levels of biological organization – from the subpopulation structure to shoot-scale morphological, physiological, and biochemical traits (Fig. 1).

### Study design

**Fig. 2:**
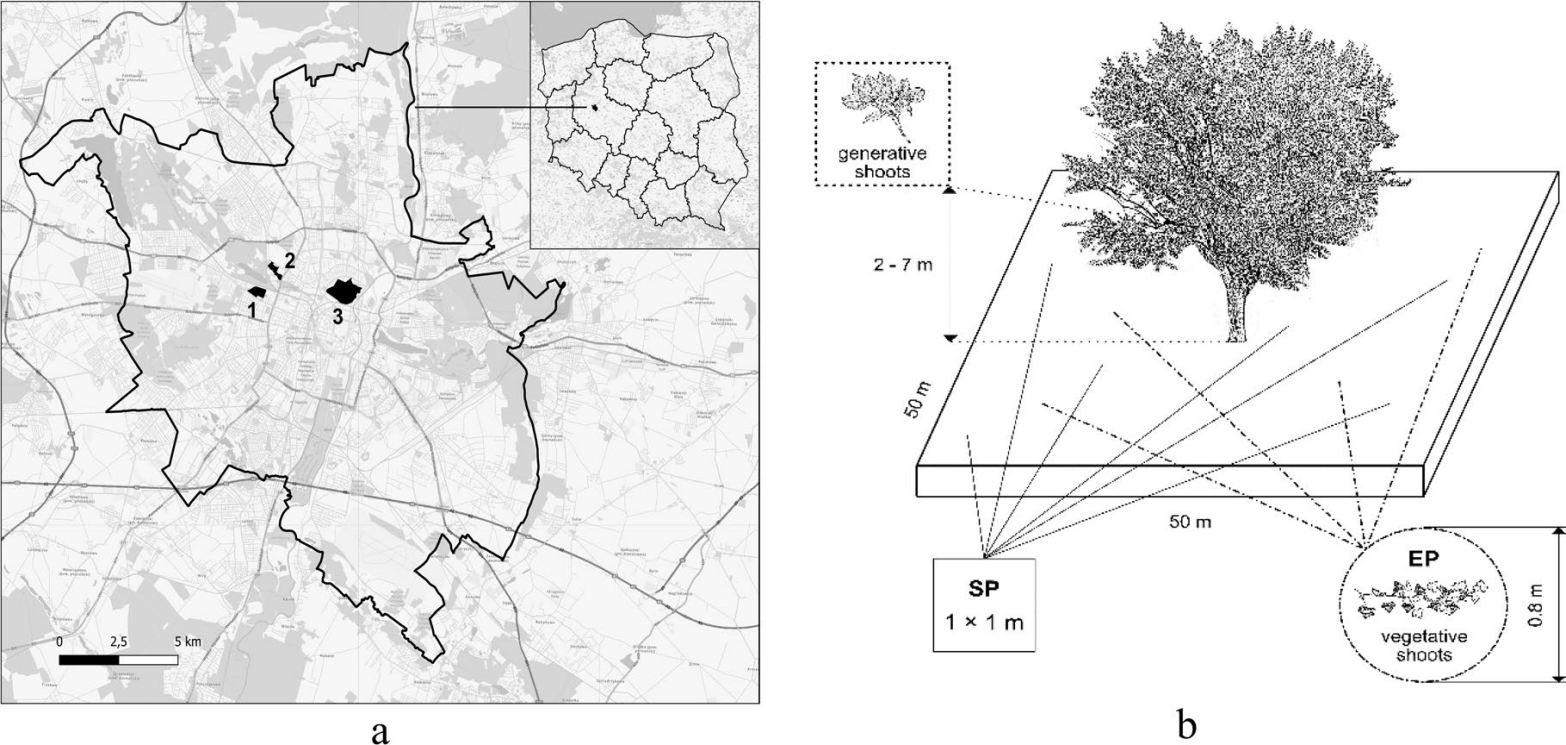
Study design: (**a**) wooded Poznan areas: 1—the Botanical Garden of Adam Mickiewicz University, 2—the Dendrological Garden of the Poznań University of Life Sciences, 3—the Citadel Park; (**b**)—split-cluster design.

The aim of the study was to compare different environmental conditions for the growth and development of vegetative/generative shoots of *H. helix* in wooded city areas. The study was conducted in May–July 2024, from 10:00 to 14:00 daily in Poznań, Central Poland (Fig. 2a). The May–July window was selected to minimize phenological variability and ensure comparability of functional traits under similar seasonal conditions. The studies were conducted in three public areas dominated by trees: 1. the Botanical Garden of Adam Mickiewicz University, 2. the Dendrological Garden of Poznań University of Life Sciences, and 3. the largest park in the city, Citadel Park (Fig. 2a). The two former gardens cover areas of approximately 20 ha, whereas the latter covers approximately 100 ha. Two clusters (50 × 50 m) were established in each of the three public areas. This cluster design enables the exploration of *H. helix* responses across a continuum of urban microhabitats differing in soil moisture and light availability, in accordance with ecological sampling strategies for environmental heterogeneity^64,65^. Consequently, six clusters under urban conditions were established (Fig. 2b). For each cluster (50 × 50 m), environmental parameters such as the volumetric water content (VWC), temperature (t), electrical conductivity (ES), and daily light integral (DLI) were measured in six randomly located subplots (SPs, 1.0 × 1.0 m) (Fig. 2b). These environmental data were averaged per cluster and treated as a fixed abiotic background for all individuals sampled within that locus, following the ecological stratification approach proposed for trait–environment analyses^66^. Additionally, four experimental plots (EPs, 0.8 × 0.8 m) were established within each cluster for the collection of morphological, functional, and biochemical data from *H. helix* shoots (Fig. 2b). This design facilitates the integration of spatial and physiological variation and supports further analyses stratified by shoot type (vegetative vs. generative) and leaf condition (healthy vs. damaged), consistent with the approaches discussed by Cornelissen et al.^65^ and Messier et al.^66^.

Field sampling, including limited destructive collection of leaf and shoot material, was conducted with permission from the managing authorities of all study sites: the Botanical Garden of Adam Mickiewicz University, the Dendrological Garden of the Poznań University of Life Sciences, and the Citadel Park in Poznań. All collections complied with institutional, local, and national regulations, and no protected or endangered species were sampled.

### Abiotic measurements

Photosynthetically active radiation (PAR) was measured using a Quantitherm PAR sensor (Hansatech Instruments) operating in the 400–700 nm waveband. Measurements were taken in the four corners and the center of each experimental plot (EP) at a height of 10 cm above the ivy canopy. The air temperature was recorded in parallel using shielded sensors to avoid direct solar radiation and rainfall interference. The daily light integral (DLI), which represents the total amount of PAR received per day (mol × m⁻^2^ × d⁻^1^), was estimated from the instantaneous PAR.

The soil volumetric water content (VWC), electrical conductivity (ES), and soil temperature (t) were measured within each 1 × 1 m subplot (SP) using a TDR-350 probe (Spectrum Technologies Inc., USA). Each SP was measured in five replicates, and mean values were computed to represent local abiotic conditions. In accordance with standard functional trait measurement protocols^67^, these environmental variables were averaged per cluster and treated as fixed abiotic background values for all individual plants sampled within the same spatial unit.

In addition to the abiotic measurements, the dominant species of the herbaceous and tree layers were recorded in each cluster. The plant species names were verified according to World Flora Online^68^. The results provide an ecological background for interpreting microhabitat variability (Table 1).

**Table 1:** Abiotic characteristics (means ± SDs) and dominant vegetation species in the studied clusters.

Wooded Poznan area*. Cl	VWC (%)	T (°C)	ES (ms × cm^-1^)	DLI (mol × m^−2^ × d^−1^)	Dominant species of herb layer	Dominant species of tree layer
1.1	8.43 ± 4.02	19.38 ± 0.64	0.68 ± 0.18	7.63 ± 3.46	*Aegopodium podagraria* L. *Impatiens parviflora* DC. *Bryonia dioica* Jacq. *Chelidonium majus* L. *Elytrigia repens* (L.) Nevski. *Lamium galeobdolon* (L.) L. *Geum urbanum* L. *Poa nemoralis* L. *Urtica dioica* L.	*Acer platanoides* L. *Acer pseudoplatanus* L. *Carpinus betulus* L. *Fraxinus excelsior* L.
1.2	7.53 ± 4.56	19.44 ± 0.83	0.62 ± 0.13	10.54 ± 4.01	*Bryonia dioica* Jacq. *Chelidonium majus* L. *Impatiens parviflora* DC. *Geum urbanum* L. *Poa nemoralis.* *Urtica dioica* L.	*Acer platanoides* L. *Acer pseudoplatanus* L. *Acer campestre* L. *Carpinus betulus* L. *Ulmus laevis* Pall.
2.3	7.94 ± 5.68	20.65 ± 1.17	0.69 ± 0.22	11.25 ± 6.56	*Elytrigia repens* (L.) Nevski. *Impatiens parviflora* DC. *Geranium robertianum* L. *Geum urbanum* L. *Filipendula vulgaris* Moench. *Urtica dioica* L.	*Acer platanoides* L. *Populus alba* L. *Carpinus betulus* L. *Fraxinus excelsior* L.
2.4	8.79 ± 5.75	19.02 ± 1.01	0.71 ± 0.35	12.38 ± 5.39	*Aegopodium podagraria* L. *Allium ursinum* L. *Filipendula vulgaris* Moench. *Geum urbanum* L. *Mercurialis perennis* L. *Pteridium aquilinum* (L.) Kuhn.	*Acer platanoides* L. *Carpinus betulus* L. *Fraxinus excelsior* L. *Populus alba* L. *Taxus baccata* L. *Ulmus laevis* Pall.
3.5	7.14 ± 3.21	20.08 ± 0.57	0.51 ± 0.25	13.88 ± 3.52	*Brachypodium sylvaticum* (Huds.) P. Beauv. *Chelidonium majus* L. *Elytrigia repens* (L.) Nevski. *Geranium robertianum* L. *Impatiens parviflora* DC. *Lamium maculatum* L.	*Acer platanoides* L. *Acer campestre* L. *Tilia cordata* Mill. *Ulmus laevis* Pall.
3.6	7.65 ± 4.99	21.08 ± 0.75	0.65 ± 0.19	12.05 ± 4.22	*Brachypodium sylvaticum* (Huds.) P. Beauv. *Bromus sterilis* (L.) Scop. *Chelidonium majus* L. *Geum urbanum* L. *Impatiens parviflora* DC. *Lamium maculatum* L. *Poa nemoralis* L. *Stenactis annua* (L.) Nees.	*Aesculus hippocastanum* L. *Acer platanoides* L. *Acer campestre* L. *Carpinus betulus* L. *Robinia pseudoacacia* L. *Ulmus laevis* Pall.

Wooded Poznan areas include (1) the Botanical Garden of Adam Mickiewicz University, (2) the Dendrological Garden of Poznań University of Life Sciences, and (3) the Citadel Park.

### Visual classification of *H. helix* leaf damage

**Fig. 3:**
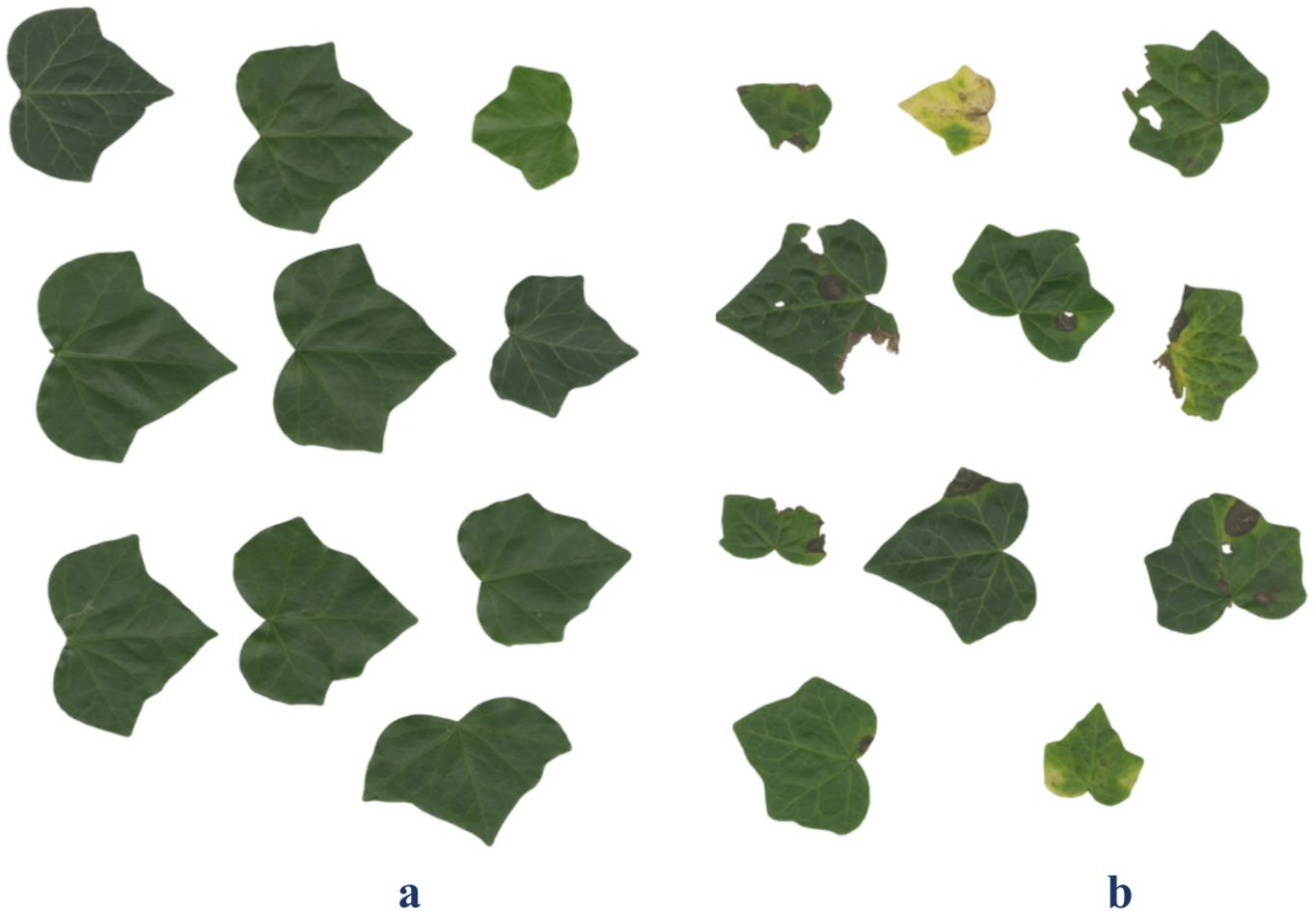
Visual comparison of healthy and damaged leaves of *H. helix* collected from vegetative and generative shoots. Panel a shows intact, physiologically healthy leaves; panel b demonstrates leaves with visible signs of damage, including necrotic spots, chlorosis, marginal drying, and biotic perforation, predominantly associated with excessive irradiance and biotic stressors.

Prior to sample collection for measurements, a visual assessment of the leaf condition was conducted, and all the leaves were classified into two categories: (1) healthy—exhibiting no visible morphological damage and showing typical coloration—and (2) damaged—exhibiting visible signs of stress. The morphological criteria for damage included necrotic spots, marginal browning, interveinal chlorosis, asymmetrical tissue bleaching of the lamina, and mechanical injuries (Fig. 3). The observed symptoms correspond to typical leaf responses to abiotic (notably excessive light) and biotic stress factors^69^. The objective of this classification was not to determine the etiology of the damage but rather to stratify the sample based on the leaf functional status, enabling a further comparative analysis of the morphological and physiological traits. Such stratification is a widely applied practice in ecological studies of urban flora, where visual damage markers serve as rapid diagnostic tools for detecting stress responses in heterogeneous environments^70^.

### Subpopulation analysis

The density of *H. helix* subpopulations was assessed within each EP, with four replicates per cluster. Subpopulation differentiation was confirmed by integrating abiotic environmental parameters (VWC, t, ES, and DLI) with the floristic composition of each cluster, as detailed in Table 1. These variables provide the ecological context for interpreting individual-level variation in morphofunctional traits. Within each EP, the number of individuals bearing vegetative shoots was recorded, whereas generative shoots were documented on host trees within the same locus, including their height range (2–7 m). The orientation of the shoots (illuminated vs. shaded side of the trunk) was also considered to capture the spatial distribution patterns of the reproductive structures along the light microgradient. The proportions of vegetative and generative shoots were calculated for each cluster and used as an indicator of the ontogenetic stage of the subpopulation. This approach aligns with the functional ecological sampling protocols proposed by Cornelissen et al.^65^.

### Biotic measurements

For each EP, vegetative and generative shoots of H. helix were selected and classified according to the leaf health status (healthy/damaged). Morphological and biometric traits were measured using fresh material; however, all biomass-related variables used in the statistical analysis were standardized to dry weight after drying at 60 °C for 48 h to avoid bias from the water content.

### Biomass measurements

A representative subset of liana individuals was sampled in the study area following the methods outlined by Condit^71^. The aboveground biomass included both leaves and stems. We used the destructive method for estimating the plant biomass. Aboveground plant organs were separated using pruning shears, tagged in the laboratory, and weighed. The collected plant material (leaves, stems) of *H. helix* individuals was dried at 65 °C in an oven (ULE 600 or UF450, Memmert GmbH + Co. KG, Germany) using standard protocols^67^. All dry biomass samples of *H. helix* were weighed. The dry biomass data were recorded to the nearest 0.01 g to ensure the accuracy of trait standardization.

### Morphological measurements

Freshly harvested *H. helix* shoots from each EP were used for the morphometric analysis. From each cluster, 10–15 individuals were randomly selected. For each shoot, ten fully expanded leaves were measured for petiole length (L_p_), leaf length (L_l_), and width (W_l_) using digital calipers (± 0.01 mm). Measurements were performed on fresh material within 2 h of harvest to ensure tissue integrity. Morphometry of generative shoots was conducted at heights of 2–7 m on both the sun-exposed and shaded sides of tree trunks. The measurement protocols followed standard trait guidelines^67^.

Phenotypic plasticity index. We quantified the degree of phenotypic plasticity in the morphological traits by calculating the phenotypic plasticity index (PPI) for each morphometric variable (L_p_, L_l_, W_l_) using the approach proposed by Valladares et al.^72^ and Nicotra et al.^73^. The index was computed as follows:

$$PPI_{x} = \frac{{\max \left( {\overline{x}} \right) - \min \left( {{\overline{\text{x}}}} \right)}}{{\max \left( {{\overline{\text{x}}}} \right)}}$$

where ${\overline{\text{x}}}$ is the mean value of a given trait in each compared group and max (${\overline{\text{x}}}$) i min (${\overline{\text{x}}}$) refer to the highest and lowest value group means, respectively.

The PPI was calculated separately for two pairwise comparisons: (1) healthy vs. damaged leaves and (2) vegetative vs. generative shoots. A higher PPI value indicates a greater degree of trait plasticity and potential adaptability to heterogeneous environmental conditions.

### Functional measurements

The leaf samples for the analysis of functional traits were collected on the same day. The leaves of each individual were harvested, placed in plastic bags and transported to the laboratory. In the laboratory, all the samples were imaged and scanned using WinFOLIA 2013 PRO software (Regent Instruments Inc., Quebec, Canada). Based on the measurements, we calculated the leaf mass per area (LMA). We further calculated the leaf mass fraction (LMF) and specific leaf area (SLA) (Table S1). The leaf traits were calculated per individual plant^67^.

Three variables were calculated to assess the effects of water stress on lianas: the leaf water content (LWC), stem water content (STWC), and aboveground biomass water content (ABWC)^74^.

Determination of photosynthetic pigment concentrations. Chlorophyll and carotenoid concentrations were determined according to the methods described by Barnes et al.^75^. Approximately 35 mg of fresh leaves was cut into small pieces (approximately 2 mm^2^) and incubated for 4 h in 5 ml of 100% dimethyl sulfoxide (DMSO) saturated with CaCO_3_ at 60 °C. The incubation continued until the leaves lost all of their color. The absorbance of the extract was measured at 665, 648, and 470 nm with a Cary 60 UV–Vis spectrophotometer (Agilent Technologies). A solution of DMSO was used as a blank.

### Biochemical measurements

The total protein content in the leaves was determined using the Bradford assay^76^, with bovine serum albumin (BSA) used as the standard. Leaf samples (~ 100 mg fresh weight) were ground in liquid nitrogen and extracted with 1.5 mL of 50 mM sodium phosphate buffer (pH 7.0) containing 2 mM EDTA and 20% polyvinylpyrrolidone (PVP). The homogenate was filtered through two layers of cheesecloth and centrifuged at 2000×*g* for 20 min at 4 °C. The supernatant was collected, and the protein concentration was measured spectrophotometrically at 595 nm. The protein content is reported as mg × g⁻^1^ fresh weight (FW). All measurements were performed in three technical replicates and two independent biological replicates per sample.

### Integrative ecological index of the adaptive response in *H. helix*

We applied a multivariate ecological evaluation approach to assess the adaptive response of *H. helix* to environmental factors^77,78^. We developed an integrative ecological index (IEI), which represents the arithmetic mean of five analytical blocks: morphometric traits; organ water, pigment, and protein contents; and functional leaf traits.

For each analytical block, a corresponding subindex was calculated based on normalized (z score) values. Normalization was performed using the following formula:

$${Z}_{i}=\frac{{x}_{i}-\overline{x}}{\sigma }$$

where ${\text{Z}}_{\text{i}}$ is the normalized value of the i parameter, ${\text{x}}_{\text{i}}$ is the original value of the parameter, $\overline{\text{x} }$ is the sample mean, and $\upsigma$ is the standard deviation.

As an example, the Morphometric index (MI) was calculated as the mean of normalized values of petiole length (*Lp*, cm), leaf blade length (*Ll*, cm) and leaf blade width (*Wl*, cm):

$$MI=\frac{{Z}_{Lp}+{Z}_{Ll}+{Z}_{Wl}}{3}$$

Analogously, the Water Stress Index (WI) integrated relative water content parameters (LWC, STWC, ABWC); the Functional Leaf Trait Index (FI) combined SLA, LMA, and LMF; the Pigment Index (PgI) included Chl a, Chl b, and Carot; and the Protein Index (PrI) was represented by the normalized protein concentration (PC). The Water Stress Index (WI), Functional Leaf Trait Index (FI), Pigment Index (PgI), and Protein Index (PrI) were calculated analogously as the arithmetic means of their respective normalized traits. Full formulas and trait lists are provided in Table S2.

Finally, the Integrative Ecological Index (IEI) was calculated as:

$$IEE=\frac{MI+WI+FI+PgI+PrI}{5}$$

### Statistical analysis

Descriptive statistics were computed for each morphofunctional group (healthy/damaged × vegetative/generative) to assess the variability in biometric traits (leaf, stem, and petiole masses), including means, standard deviations (SD), coefficients of variation (CV), and minimum, maximum, and median values. Relationships between biometric traits and soil variables (VWC, t, and ES) were evaluated using Spearman’s rank correlation coefficient (r_s_). Prior to all parametric analyses (regressions, PCA, CDA), assumptions were verified uniformly: normality—Shapiro–Wilk test, homoscedasticity—Levene’s test, multicollinearity—variance inflation factor (VIF < 5). Statistical significance thresholds were set at p < 0.05, p < 0.01, and p < 0.001. Regression analyses (linear and polynomial) were performed separately for each trait and morphofunctional group to evaluate the effects of VWC, t, and ES on leaf functional traits (SLA, LMA, LA, LMF). Model quality was assessed using R^2^ and p-values. Principal component analysis (PCA) was used to explore broader ecological patterns. Canonical discriminant analysis (CDA) was conducted to classify samples based on pigment composition (Chl a, Chl b, Chl a + b, Carot, Chl a/b) by shoot type and leaf condition. Canonical correlation analysis (CCA) assessed multivariate relationships between abiotic environmental predictors (VWC, t, ES, DLI) and pigment traits. The analysis used standardized coefficients of the first canonical function and canonical loadings; only variables with VIF < 5 were included. Spearman’s correlations were calculated separately for each morphofunctional group (hv, dv, hg, dg) to analyze relationships between water-related leaf traits (LWC, STWC, ABWC) and abiotic factors (VWC, t, ES). Results were visualized using multipanel correlation matrices with 95% confidence ellipses and annotated r_s_ values (Table S3). One-way ANOVA was used to assess differences in protein content between shoot types after verifying variance homogeneity. Associations between protein content and DLI, VWC, t, ES, as well as relationships between protein content and pigment composition were evaluated using Spearman’s correlations due to non-normal data. Partial least squares (PLS) regression examined multivariate links between pigment composition and protein content in *H. helix* leaves. VIP scores (VIP > 1.0) identified the most influential predictors. Model quality was based on the variance explained by the first two latent factors for both predictor (X) and response (Y) variables. Finally, the relationship between protein content and SLA was assessed using Spearman’s correlations (non-normal distributions) and visualized with a second-order polynomial regression to illustrate trends.

All the statistical analyses and visualizations were performed using OriginPro 2024 (OriginLab Corporation, Northampton, MA, USA).

## Results

The results are presented sequentially: subpopulation structure and local environmental characteristics, morphometric and biometric traits, impacts of soil parameters and light intensity on functional leaf traits and pigments variation, water status indicators and their relationships with pigments and soil factors, protein content variability and environmental drivers, and the integrative ecological index across shoot type and leaf condition.

### Subpopulation structure of *H. helix* and environmental characteristics of the study sites

The abiotic characteristics of the six investigated sites that shape the development conditions of *H. helix* subpopulations within urban parks differed. Across the studied gradients of the soil volumetric water content (VWC), electrical conductivity (ES), soil temperature (t), and daily light integral (DLI), a transition was observed from dry, sparsely shaded areas with a rapidly warming substrate to moderately moist sites characterized by well-developed tree canopies providing significant shading (Table 1).

The highest soil temperatures were recorded at sites with the lowest VWC, indicating a typical pattern in urban environments, where low soil moisture is associated with rapid diurnal heating. As DLI increased and VWC decreased, soil temperature approached the upper thermal tolerance limits of the ivy root system. In contrast, the soil electrical conductivity remained within a moderate range, suggesting relatively neutral soil conditions with respect to salinity. The *H. helix* subpopulations studied were distributed along this environmental gradient, which must be considered when interpreting functional trait responses.

The composition of the tree and herbaceous layers confirmed the presence of both typical shade-tolerant species of urban forest parks and species with broad ecological amplitudes capable of withstanding moisture and light fluctuations. In clusters with the highest soil moisture (Clusters 1 and 4), shade-tolerant nemoral species (e.g., *A. podagraria* and *F. excelsior*) predominated, whereas in more light-exposed conditions (Clusters 5 and 6), light-demanding species and ruderal urbanophytic taxa (e.g., *B. sterilis*, *P. nemoralis*, and *A. hippocastanum*) were more common.

Within each cluster, a clear structural organization of *H. helix* subpopulations was observed, reflecting the interplay between shoot morphotype composition and site-specific environmental gradients. For each EP within all the clusters, an average of 3–4 individuals bearing vegetative shoots was recorded. Generative shoots were consistently observed climbing the trunks of the dominant tree species at heights ranging from 2 to 7 m, with the highest shoot densities (7–10 shoots per tree) occurring in the upper, sun-exposed crown zones. A consistent 40.55% reduction in the leaf density of generative shoots was recorded at the mid-canopy height (2–3 m) compared with the upper canopy (7 m), and the proportion of damaged leaves on generative shoots decreased by 25.35–34.13% in shaded and semishaded conditions relative to high-light sites.

### Morphometric and biometric characteristics of *H. helix* shoots

The analysis of the biometric and morphometric parameters of *H. helix* revealed distinct differences between vegetative and generative shoots, as well as between healthy and damaged leaves (Table S4). The highest fresh leaf mass (L_m_) was recorded in healthy leaves of vegetative shoots (102.62 ± 24.55 g). In contrast, healthy leaves on generative shoots presented nearly twofold lower average values (48.00 ± 36.03 g), accompanied by a markedly higher coefficient of variation than the relatively stable leaf mass observed in vegetative shoots (Table S4). The fresh mass of damaged leaves was substantially lower than that of healthy leaves in both shoot types. A similar trend was observed for the dry leaf mass: the highest values were recorded in healthy vegetative leaves (32.28 ± 7.72 g), whereas the lowest occurred in damaged leaves of generative shoots (2.72 ± 2.05 g). The stem mass (S_m_) also varied considerably across groups. Vegetative shoots bearing healthy leaves presented the highest values (151.11 ± 75.67 g), whereas generative shoots with damaged leaves presented a markedly reduced biomass, with a consistently high degree of variability (0.86). A comparable pattern was found for the leaf petiole mass (L_pm_), with healthy leaves having the highest values and damaged generative leaves having the lowest values. Overall, leaf damage was associated with decreases in most biometric traits. However, vegetative shoots demonstrated greater structural consistency even in the presence of foliar damage. The morphometric traits of *H. helix* leaves presented moderate intergroup variability, with distinct differences observed between generative and vegetative shoots, as well as between healthy and damaged leaves. The greatest mean petiole length (L_p_) was recorded in healthy leaves of generative shoots (11.21 cm), whereas the lowest values were observed in healthy leaves of vegetative shoots (10.33 cm) (Table S4). Compared with vegetative shoots, generative shoots, regardless of the leaf condition, presented higher values for the leaf blade length (L_l_). In contrast, the highest values of leaf blade width (W_l_) were found in healthy leaves of vegetative shoots.

Among the analyzed traits, leaf blade length (L_l_) presented the highest degree of ecological plasticity (PPI = 0.244). Intermediate plasticity was observed for leaf width (W_l_; PPI = 0.116), whereas the petiole length (L_p_) had the lowest plasticity index (PPI = 0.079).

The correlation analysis (Spearman’s rank) between the biometric traits of *H. helix* shoots and environmental parameters revealed several statistically significant associations. Soil temperature (t) was moderately and positively correlated with both the fresh leaf mass (r_s_ = 0.24, p < 0.01) and dry leaf mass (r_s_ = 0.22, p < 0.01) in vegetative shoots. The soil electrical conductivity (ES) also exhibited moderate positive correlations with vegetative leaf mass in both the fresh (r_s_ = 0.21, p < 0.01) and dry states (r_s_ = 0.23, p < 0.01). Significant negative correlations were found between daily light integral (DLI) and both fresh leaf mass (r_s_ = -0.30, p < 0.01) and petiole mass (r_s_ = -0.28, p < 0.01) in generative shoots. In contrast, soil volumetric water content (VWC) exhibited a moderately positive, yet less pronounced, association with fresh leaf mass in vegetative shoots (r_s_ = 0.14, p < 0.05).

### Impacts of soil parameters (VWC, ES, and t) on the functional leaf traits of *H. helix* shoots

**Fig. 4:**
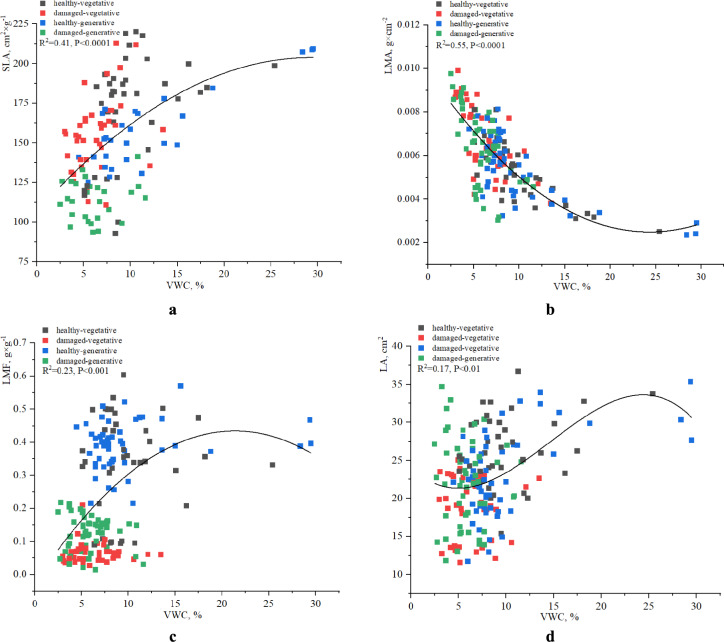
Relationships between soil volumetric water content (VWC, %) and functional leaf traits of *H. helix* across different shoot types and leaf conditions: (**a)** SLA, (**b**) LMA, (**c**) LMF, (**d**) LA.

Given that soil moisture is a key limiting factor for the distribution and development of *H. helix*, the analysis of functional leaf traits was conducted using second-order polynomial regression models separately for each parameter (SLA, LMA, LA, and LMF) (Fig. 4).

A moderately positive relationship was identified between the specific leaf area (SLA) and volumetric water content (VWC) (R^2^ = 0.41, p < 0.0001). The SLA increased with increasing soil moisture, reflecting an adaptive tendency toward the development of thinner leaves with larger surface areas in the presence of improved water availability. Compared with their counterparts with damaged leaves, generative shoots bearing healthy leaves presented higher SLA values, particularly in the presence of an elevated VWC. In contrast, vegetative shoots with damaged leaves presented a reduction in the SLA, regardless of the soil moisture content (Fig. 4a).

LMA exhibited a clear inverse relationship with VWC, as confirmed by a third-order polynomial model with a high coefficient of determination (R^2^ = 0.55, p < 0.0001). As VWC increased, LMA values sharply decreased within the range of up to ~ 10% soil moisture and subsequently stabilized at lower levels. This pattern suggests the development of less dense leaf structures under more humid conditions. Healthy leaves of vegetative shoots presented the highest LMA values across the dataset, particularly under low VWC conditions (< 10%). Damaged leaves on vegetative shoots presented lower LMA values. Healthy leaves of generative shoots presented a relatively stable LMA across a wider VWC range, whereas damaged generative leaves presented greater dispersion and a decreasing trend in LMA with an increasing VWC, albeit with a less pronounced slope (Fig. 4b).

LMF showed a weak to moderate nonlinear relationship with the VWC, described by a polynomial model (R^2^ = 0.23, p < 0.001). As VWC increased to ~ 18–20%, LMF also increased, reaching a peak and subsequently decreasing. This decline may reflect overhydration or secondary stress effects. The highest LMF values were observed in healthy leaves of generative shoots at an intermediate to high VWC (15–25%). Leaf damage was associated with a lower LMF across all shoot types, particularly in vegetative shoots (Fig. 4c).

Analysis of the relationship between LA and VWC revealed a statistically significant but weak polynomial relationship (R^2^ = 0.17, p < 0.01). The model curve was moderately parabolic, showing a trend of increasing LA within the VWC range of approximately 10–25%. At VWC levels below 10%, lower LA values predominated. After reaching peak LA values at approximately 25% VWC, no further clear trend was observed. Healthy leaves of generative shoots generally presented larger leaf areas in the intermediate-to-high VWC range. Vegetative shoots with damaged leaves consistently developed smaller leaves, regardless of the soil moisture conditions. Healthy vegetative leaves were characterized by a broader range of LA values across the full moisture gradient. In contrast, damaged generative leaves presented moderate variability in LA but no distinct relationship with VWC (Fig. 4d).

Considering additional key ecological drivers is also important to understand the multivariate structuring of environmental variables such as soil temperature (t) and electrical conductivity (ES) in relation to functional leaf traits, as assessed using principal component analysis (PCA).

**Fig. 5:**
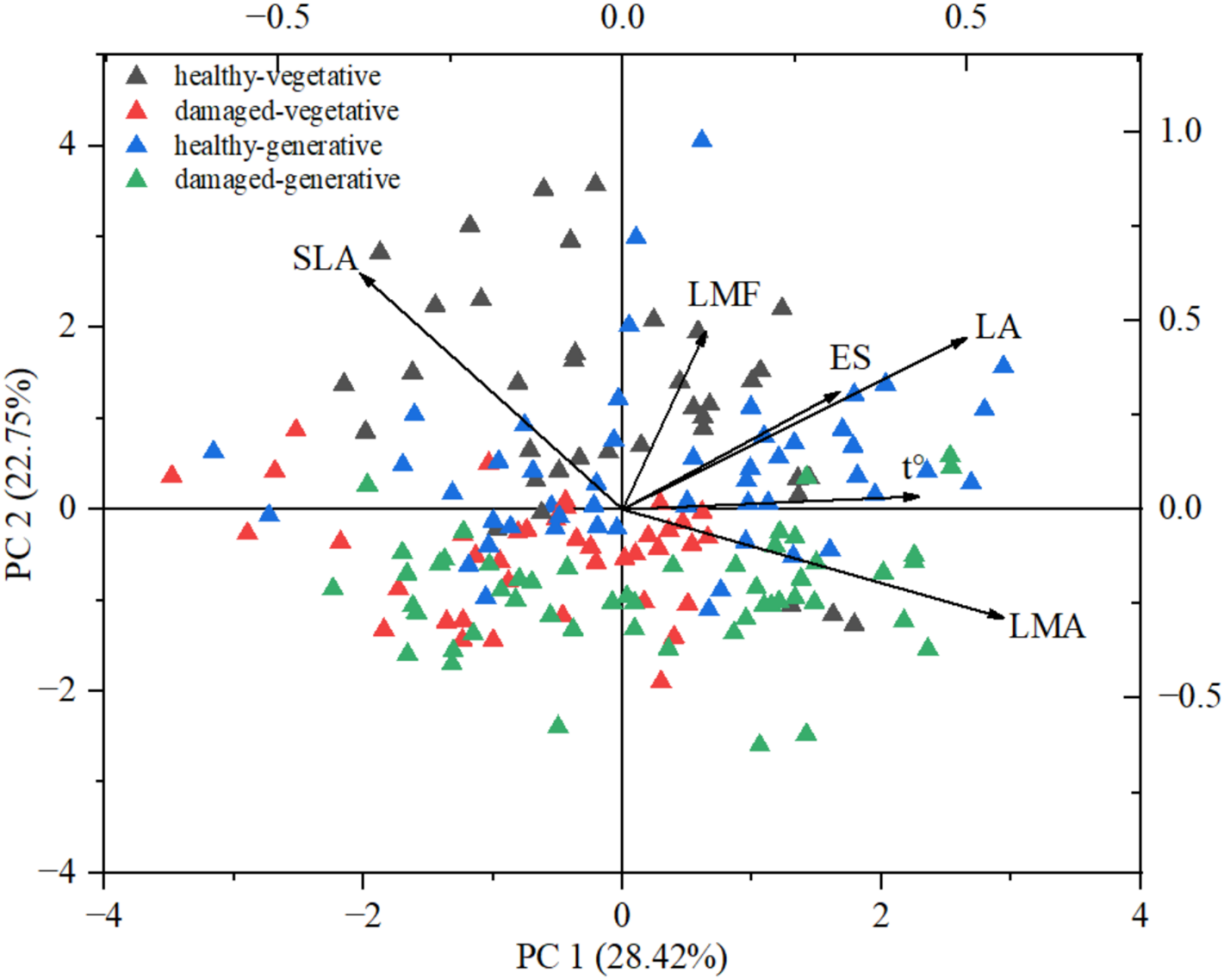
Biplot of principal component analysis (PCA) based on functional leaf traits (SLA, LA, LMF, LMA) and soil parameters (t, ES) in *H. helix*.

The first two principal components (PC1 and PC2) together explained 51.17% of the total variance in the combined dataset of functional and abiotic variables, with PC1 accounting for 28.42% and PC2 accounting for 22.75% of the variation (Fig. 5). PC1 was associated primarily with variables such as LA, t, and ES, indicating predominant influences of soil thermal and ionic conditions on leaf expansion. PC2 reflected variations in the SLA and LMF, capturing the functional response of leaves to the soil water regime. The spatial distribution of the sample points, classified by shoot type and leaf condition, indicated partial structuring in the component space. Healthy leaves from generative shoots were predominantly located in the quadrant with positive PC1 values, suggesting an association with larger LAs and potentially more favorable conditions (i.e., higher t and ES). In contrast, damaged generative leaves tended to cluster in the lower ranges of both PC1 and PC2, which was associated with higher LMA values. Leaves from vegetative shoots were more evenly distributed near the plot center and exhibited less pronounced clustering. The vector for SLA was oriented in the opposite direction to those for LA, t, and ES, confirming the inverse relationship between SLA and these environmental parameters. Vectors for LA, ES, and t were aligned, suggesting a concerted influence of these abiotic drivers. The vector for LMA was positioned along the negative axis of PC1, indicating its distinct contribution to the variation in leaf traits. Overall, the PCA visualizations facilitate the integration of functional traits into a multivariate framework of *H. helix* responses to soil-related environmental gradients, offering valuable insights into the adaptive strategies of the species.

### Impact of light intensity (DLI) on the functional leaf traits of *H. helix* shoots

#### Relationship between DLI and pigment content in leaves

**Fig. 6:**
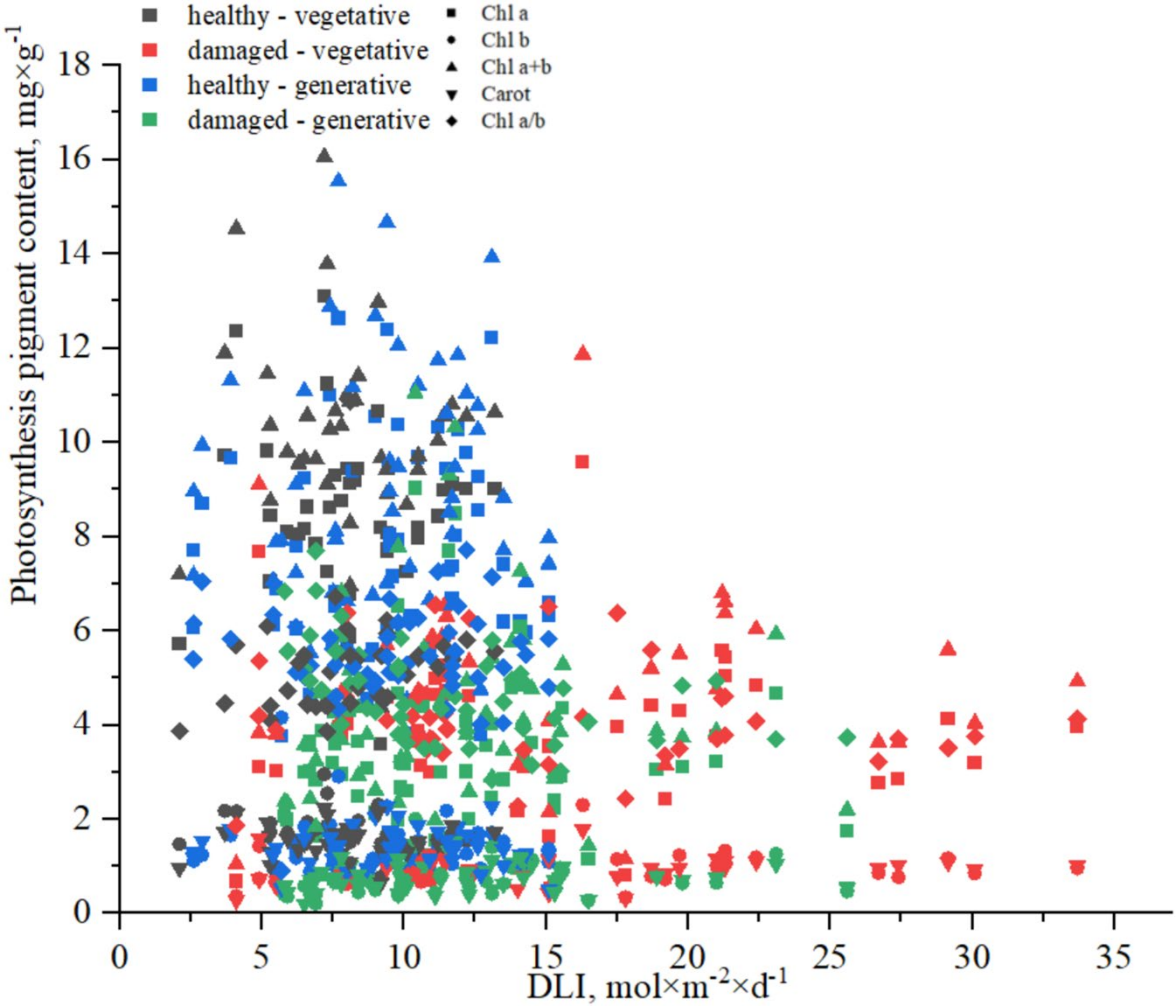
Pigment content in *H. helix* leaves across a gradient of DLI. The concentrations of Chl a, Chl b, Chl a + b, Carot, and Chl a/b ratio are shown for vegetative and generative shoots with healthy and damaged leaves.

Analysis of the photosynthetic pigment content in *H. helix* leaves revealed a nonlinear response to changes in DLI (Fig. 6). Within the range of DLI values up to approximately 15 mol × m⁻^2^ × d⁻^1^, concentrations of Chl a, Chl b, total chlorophyll (Chl a + b), and carotenoids (Carot) remained relatively stable, indicating high within-group variability. However, as DLI increased above ~ 15–18 mol × m⁻^2^ × d⁻^1^, a consistent decrease in pigment concentration was observed, potentially indicating the occurrence of photodestructive effects or disrupted pigment regulation in response to excessive irradiance. The most pronounced reductions were noted for Chl a + b, Chl a, and Chl b contents. Compared with chlorophylls, carotenoids followed a similar trend but with a more gradual decrease. The chlorophyll a/b ratio (Chl a/b) did not clearly depend on the DLI, with its values fluctuating within a narrow range, possibly reflecting the maintenance of a functional balance between photosystems and the absence of major restructuring in antenna complexes.

Within each shoot type and leaf condition, a trend toward lower pigment concentrations was observed in damaged leaves, particularly at DLI levels exceeding 20 mol × m⁻^2^ × d⁻^1^. Generative shoots bearing healthy leaves presented the highest pigment profiles within the intermediate DLI range.

### Canonical discriminant analysis of leaf pigment profiles

**Fig. 7:**
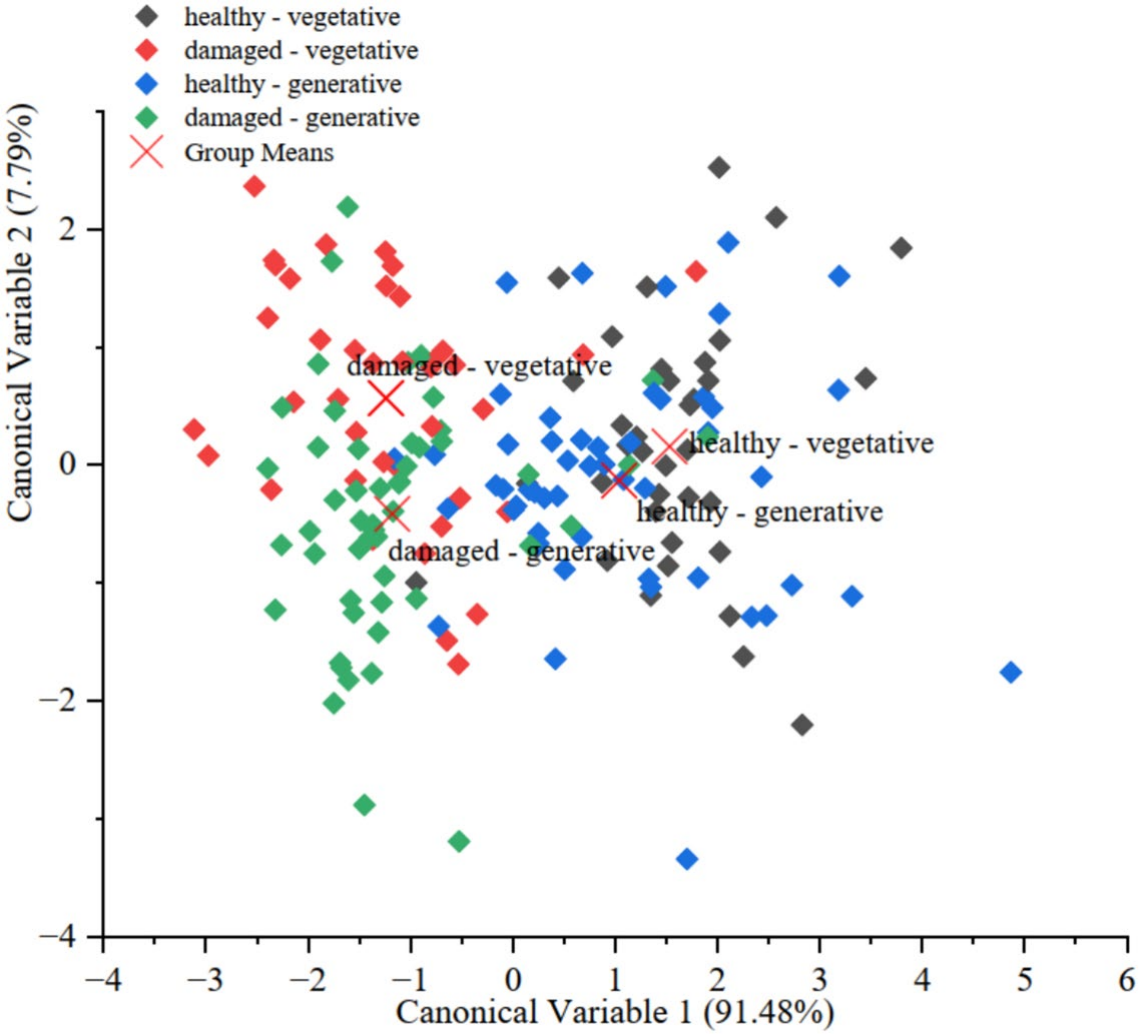
Canonical score plot resulting from canonical discriminant analysis (CDA) of pigment traits (Chl a, Chl b, Chl a + b, Carot, Chl a/b). Group centroids are marked as crosses ( ×). Groups: healthy/vegetative, damaged/vegetative, healthy/generative, and damaged/generative. Scatterplot of canonical variables CV1 and CV2 are based on pigment traits.

The Shapiro‒Wilk test revealed deviations from normality for several pigment-related variables within the predefined groups. In particular, *Chl a content*, *Chl b content*, and *Chl a/b* ratio did not follow a normal distribution in most subgroups (*p* < 0.05), especially in damaged leaves of both shoot types. In contrast, total chlorophyll (*Chl a* + *b*) and carotenoid contents were normally distributed. Despite these partial violations of the normality assumption, canonical discriminant analysis (CDA) was applied, as this method is considered robust to moderate deviations in multivariate ecological datasets, which inherently exhibit biological variability. The first canonical variable (CV1) accounted for 91.48% of the total variance between groups, whereas the second axis (CV2) explained an additional 7.79%. Overall, the first two canonical axes captured 99.26% of the total discriminatory information in the dataset (Fig. 7).

A statistically significant difference between groups was confirmed by Wilks’ lambda (0.44815, F = 3.29411, p < 0.0001), indicating the high discriminatory power of the pigment profile in differentiating the morphofunctional types of *H. helix* shoots. The canonical variable biplot revealed clear separation between groups. Healthy leaves of vegetative shoots occupied the most distant position and formed a compact cluster, reflecting their stable and homogeneous pigment composition (Fig. 7). In contrast, damaged leaves of generative shoots presented pronounced heterogeneity and a wide dispersion pattern, which may indicate disturbances in pigment metabolism or a reduced photosynthetic stability under environmental stress. The discriminant model achieved the highest classification accuracy (72.97%) for the ‘healthy–vegetative’ group. The accuracy for the other groups was lower: 61.54% for ‘damaged–vegetative’, 54.90% for ‘healthy–generative’, and 61.54% for ‘damaged–generative’ leaves. The overall classification accuracy reached 62.01%. Despite this moderate overall performance, the spatial distribution of samples in the canonical space showed the distinct clustering of healthy vegetative leaves and high variability among damaged generative leaves. These findings suggest that the pigment profile reflects functional differences between shoot types and leaf conditions. Thus, even with limited discriminatory precision, pigment parameters can be regarded as sensitive indicators of the morpho-functional status of *H. helix* shoots, particularly under urban environmental constraints.

### Relationships between the pigment traits of *H. helix* leaves and environmental factors

A canonical correlation analysis (CCA) was employed to assess the multivariate relationships between environmental predictors (VWC, ES, t, and DLI) and pigment parameters in *H. helix* leaves (Fig. S2). The first canonical component explained 34.96% of the variance in the predictor block (X) (Fig. S2a) and 15.54% of the variance in the response block (Y) (Fig. S2b). The subsequent canonical axes contributed substantially less to the explained variation. Taken together, the first four canonical components accounted for approximately 17% of the total variance in the pigment block, indicating a moderate level of multivariate association between abiotic factors and the pigment profile. Among the environmental variables, VWC, t, and DLI presented the highest canonical loadings, with DLI showing a clearly negative association with all pigment parameters, except for the Chl a/b ratio.

**Fig. 8:**
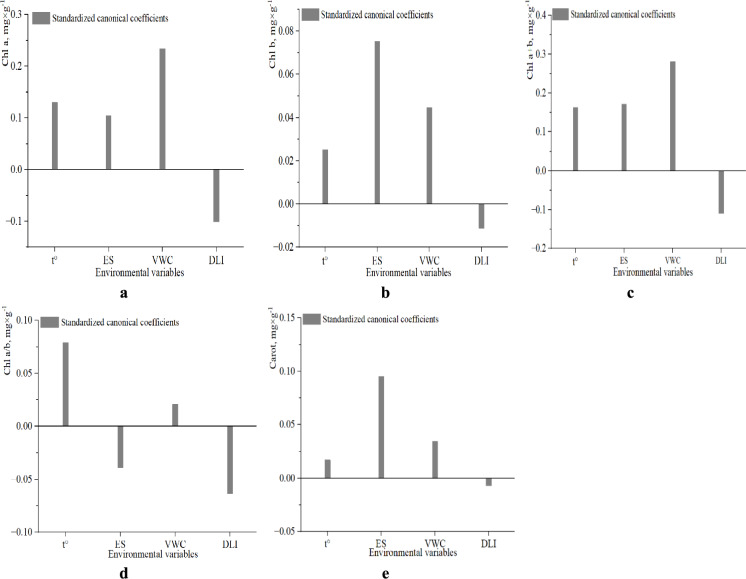
Standardized canonical coefficients from canonical correlation analysis (CCA) showing the contribution of environmental predictors to the variance in photosynthetic pigment traits in *H. helix* leaves. The diagrams illustrate the effect of soil temperature (*t*), soil electrical conductivity (ES), volumetric water content (VWC), and Daily Light Integral (DLI) on (**a**) Chl *a*, (**b**) Chl *b,* (**c**) Chl *a* + *b,* (**d**) Chl *a/b*, and (**e**) Carot. Bars represent standardized canonical coefficients along the first canonical axis.

The analysis of standardized canonical coefficients highlighted the most influential abiotic variables (Fig. 8). Chl a content showed a moderate positive association with VWC (~ 0.24), along with weaker positive contributions from t and ES (Fig. 8a). In contrast, DLI clearly had a negative effect, which was consistent with previous univariate analyses in which Chl a content decreased under a high light intensity. Chl b content displayed similarly positive coefficients for ES, VWC, and t, suggesting a combined influence of these factors on the stability of the pigment complex. DLI again showed a negative association (Fig. 8b). For the total chlorophyll content (Chl a + b), VWC was the strongest positive contributor (~ 0.28), whereas temperature and conductivity had moderate positive effects (Fig. 8c). DLI had a negative effect, supporting its role as a potential inhibitory factor under excessive light conditions. Chl a/b ratio generally displayed low sensitivity to abiotic variation, as indicated by the low magnitude of the canonical coefficients for all the predictors (Fig. 8d). The strongest, although still moderate, positive contribution was associated with temperature. Both ES and DLI exhibited weak negative coefficients. These patterns suggest relative stability in the light-harvesting complex organization of photosystems, in contrast to the more variable chlorophyll parameters. The carotenoid (Carot) content was more responsive to soil-related parameters, particularly ES and VWC, which clearly presented positive coefficients (Fig. 8e). As observed for the other pigments, DLI had a minor negative impact.

### Water status as an integral indicator of the morphofunctional response to environmental factors

Given the critical importance of water availability for the stable functioning of leaf structures, particular attention has been given to analyzing the water content in leaves (LWC) and shoots (STWC) and the total aboveground biomass (ABWC). These parameters are considered integral indicators of physiological stability, as they combine morphometric attributes with functional responses to environmental factors.

### Relationships between LWC, STWC and ABWC, and shoot type/leaf health

LWC was significantly different between the studied groups (F = 7.65, p < 0.0001). The highest mean LWC values were recorded for the healthy leaves of vegetative shoots (67.65 ± 2.61), whereas the lowest values were observed for the damaged leaves of generative shoots (61.29 ± 10.95) (Table S5). Levene’s test did not indicate violations of the homogeneity of variance (p = 0.18), thus supporting the appropriateness of using ANOVA for this parameter. STWC exhibited the most pronounced between-group variation (F = 100.27, p < 0.0001). The highest STWC was recorded in generative shoots bearing damaged leaves (77.07 ± 2.69), likely reflecting a compensatory response to impaired leaf hydration. The vegetative shoots maintained consistently high STWC levels (73.09 ± 2.94) (Table S5). Homogeneity of variance was confirmed by Levene’s test (p = 0.22). Statistically significant group differences were also found for the ABWC (F = 29.31, p < 0.0001). Levene’s test revealed no significant violations of the homogeneity of variance (p = 0.11), confirming that ANOVA was also appropriate for this trait.

### Relationships between LWC, STWC and ABWC, and pigment characteristics

The correlation analysis revealed diverse associations between hydration parameters and pigment traits across the four functional groups of *H. helix*, stratified by shoot type and leaf condition (Table 2).

**Table 2:** Spearman’s correlation coefficients between hydration parameters (LWC, STWC, and ABWC) and pigment contents within the shoot type/leaf health groups of *H. helix.*

Relationship		Group			
		Healthy vegetative	Damaged vegetative	Healthy generative	Damaged generative
LWC	Chl a	0.24*	0.13*	0.18*	0.10^°^
	Chl b	0.31**	0.11*	0.17^°^	0.07^°^
	Chl a + b	0.27*	0.13*	0.29*	0.08^°^
	Chl a/b	− 0.26*	− 0.02^°^	0.19*	0.09*
	Carot	0.22^°^	0.03^°^	0.02^°^	0.08^°^
STWC	Chl a	0.14*	− 0.09^°^	0.34***	0.02^°^
	Chl b	0.10^°^	0.17	0.18^°^	0.01^°^
	Chl a + b	0.13*	− 0.03^°^	0.26*	0.09*
	Chl a/b	0.11*	0.00^°^	0.17^°^	0.09*
	Carot	0.20*	0.01^°^	0.26**	0.09^°^
ABWC	Chl a	0.31**	− 0.03^°^	0.19*	− 0.02^°^
	Chl b	0.28*	− 0.10^°^	0.01^°^	− 0.03^°^
	Chl a + b	0.31**	− 0.07^°^	0.17^°^	− 0.02^°^
	Chl a/b	0.06^°^	0.29**	0.24*	0.01^°^
	Carot	0.34**	− 0.03^°^	0.32**	− 0.00^°^

Significance levels: ***p < 0.001, **p < 0.01, *p < 0.05, and ^°^p > 0.05.

The greatest number of statistically significant and biologically meaningful correlations was observed in the healthy leaves of vegetative shoots. In particular, ABWC was significantly correlated with Chl a, Chl b, and Carot contents, indicating strong functional integration between the water balance and the pigment system (Table 2). Additionally, a negative correlation between LWC and Chl a/b ratio (r = -0.26, p < 0.05) was detected, which may suggest the stabilization of photosystem antenna complexes under improved hydration conditions. In contrast, the damaged leaves of vegetative shoots presented mostly weak or statistically nonsignificant correlations, potentially reflecting impaired regulatory control over pigment synthesis. In healthy leaves of generative shoots, hydration parameters, especially STWC and ABWC, presented moderate positive associations with the total chlorophyll (Chl a + b) and carotenoid contents (up to r = 0.34, p < 0.001) (Table 2). Moreover, in the damaged leaves of generative shoots, the relationships between hydration and pigment traits were weak and statistically insignificant.

### Relationships between hydration parameters and soil environment characteristics (VWC, ES, and t)

**Fig. 9:**
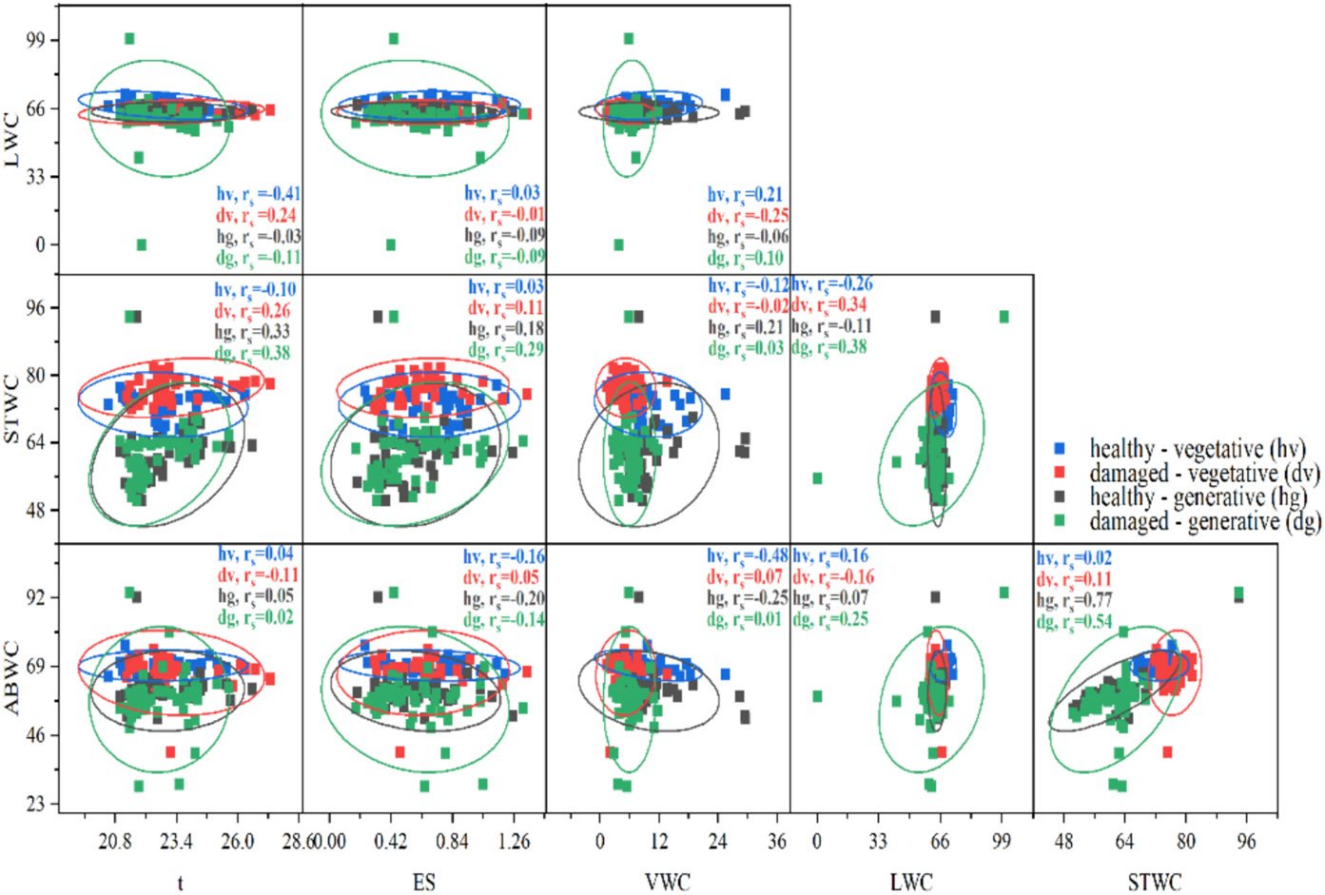
Pairwise correlations between abiotic factors (t, ES, VWC) and hydration parameters (LWC, STWC, ABWC) in four experimental groups: healthy vegetative (hv), damaged vegetative (dv), healthy generative (hg), and damaged generative (dg) shoots of H. helix*. Ellipses represent 95% confidence intervals. Spearman’s correlation coefficients (r) are shown for each group and pairwise comparison. Note: Some panels were excluded from the correlation matrix as the analysis focused on the relationship between environmental factors and leaf hydration traits rather than intercorrelations among predictors.

**Fig. 10:**
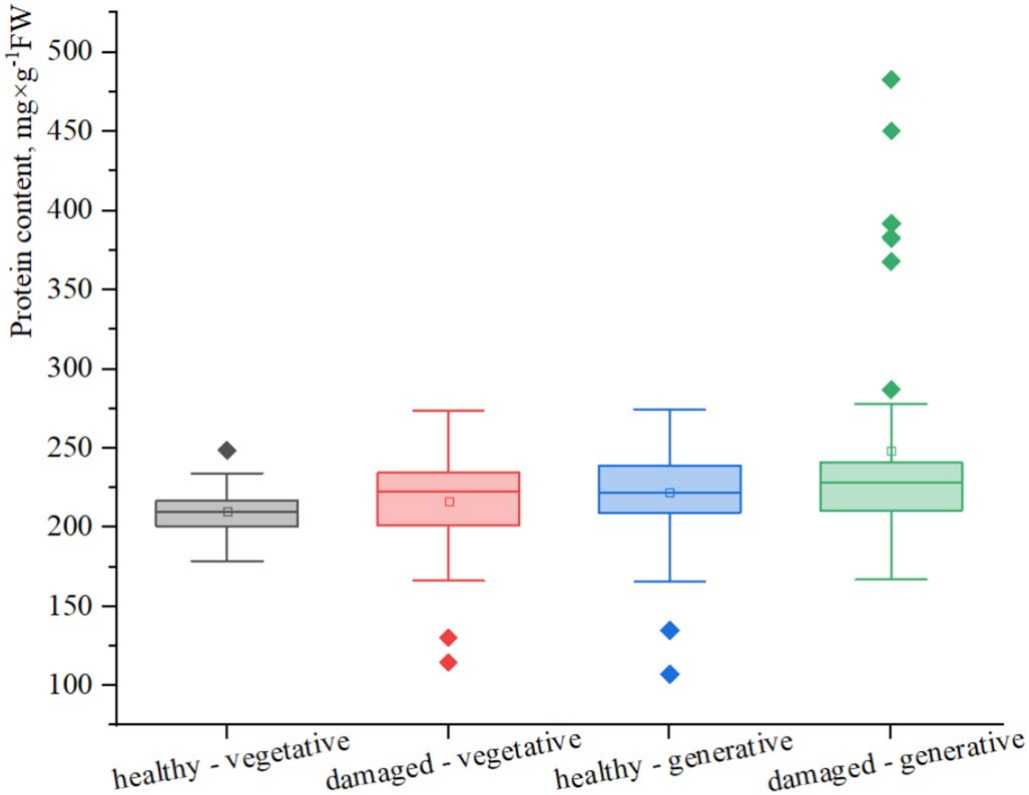
Protein content (mg × g^−1^FW) in groups of *H. helix* shoots.

Correlation analysis (Fig. 9) revealed distinct patterns of associations between abiotic environmental factors (VWC, ES, and t) and shoot hydration parameters of *H. helix* (LWC, STWC, and ABWC) across the four functional groups. Detailed *p* values are provided in Table S3. Pairwise comparisons among the environmental predictors themselves (e.g., ES vs. t) were excluded from the analysis. Moderate positive correlations between VWC and LWC were observed in the leaves of vegetative shoots (hv: *r*_*s*_ = 0.21, dv: *r*_*s*_ = 0.25), indicating a degree of hydration responsiveness to soil water availability (Fig. 9). This relationship was notably weaker in generative shoots, suggesting a potential loss of regulatory dependence at this developmental stage. A similar trend was evident for ABWC: the strongest positive association with VWC was recorded in hv (*r*_*s*_ = 0.48), whereas in hg, the correlation was almost twice as low (*r*_*s*_ = 0.25) (Fig. 10). Soil temperature exhibited the strongest positive correlations with STWC in dv (*r*_*s*_ = 0.26) and hg (*r*_*s*_ = 0.33), with the highest value observed in dg (*r*_*s*_ = 0.38). A notable positive correlation was also found between LWC and soil temperature in healthy vegetative leaves (*r*_*s*_ = 0.41). Similarly, ES displayed a single moderate positive association with STWC in dg (*r*_*s*_ = 0.29), possibly reflecting the influence of soil mineral composition on tissue osmotic properties.

### Ecophysiological variability and environmental drivers of the protein content in *H. helix* leaves

Protein content is a key indicator of the physiological status of plants and reflects both functional specialization and environmental responses. In *H. helix*, the variability in leaf protein concentration may indicate diverse adaptation strategies to heterogeneous ecological conditions.

### Changes in protein content in *H. helix* leaves, depending on the shoot type and leaf condition

ANOVA results revealed significant differences in protein content among the groups (Table 3). The lowest mean protein content was observed in the healthy vegetative group (*hv*) (210.08 mg × g^−1^FW), whereas the highest mean protein content was recorded in the damaged generative group (*dg*) (248.14 mg × g^−1^FW), likely reflecting a stress-related response, where damaged generative leaves engage in stronger recovery mechanisms.

**Table 3:** Results of one-way ANOVA of the protein content in *H. helix* shoots.

Group	Mean	SD	SE of the mean	Variance	F	p value
Healthy vegetative (hv)	210.08	14.62	2.40	213.89	6.84	< 0.0001
Damaged vegetative (dv)	216.13	32.33	5.17	1045.45		
Healthy generative (hg)	222.36	30.09	4.21	905.71		
Damaged generative (dg)	248.14	68.95	9.56	4753.87		

Additionally, the *dg* group presented the highest standard deviation (68.95), indicating considerable variability within this group.

The lowest variability was observed in healthy vegetative leaves. The damaged leaves of both shoot types presented high variability, with the maximum variance recorded for the damaged generative group (*dg*) (4753.87). The F value and p values indicated statistically significant differences in protein contents among the groups. The statistical analysis also revealed the presence of nonrandom individual outliers in the protein content within each group (Fig. 10).

The *hv* group presented the smallest range and variability, confirming its greater stability in response to environmental conditions. In contrast, the *dg* group presented not only the highest median protein content but also the widest range of values.

### Relationships between protein contents in *H. helix* leaves and environmental factors

Relationships between the protein contents in *H. helix* leaves and environmental factors (DLI, VWC, t, and ES) were assessed using Spearman’s rank correlation coefficient.

No statistically significant correlations were detected between the protein content and any of the tested environmental variables. The strongest, yet still nonsignificant, relationship was a weak negative correlation between protein content and ES (r_s_ = − 0.11, p > 0.05). The correlations with other factors were even weaker: t, r_s_ = − 0.10, p > 0.05; VWC, r_s_ = 0.09, p > 0.05; and DLI, r_s_ = 0.01, p > 0.05. These results suggest that under the current sampling framework, the abiotic factors examined have minimal influence on protein accumulation in leaves.

### Relationships between protein content and pigment traits in *H. helix* leaves

**Fig. 11:**
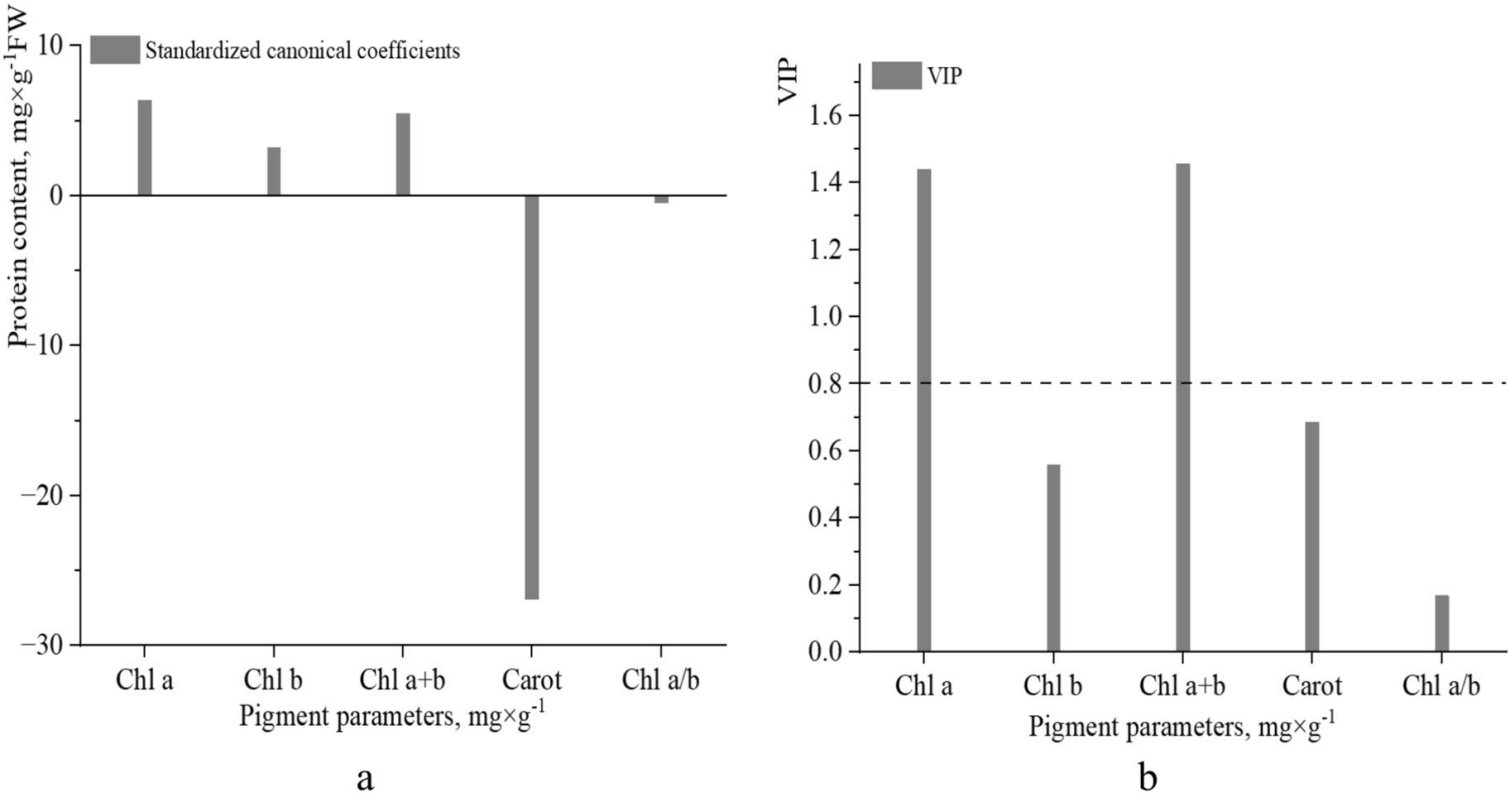
Pigment traits in predicting protein content (mg × g⁻^1^ FW) in *H. helix* leaves based on the PLS regression model: (**a**) standardized canonical coefficients illustrating the direction and strength of the relationship; (**b**) variable importance in projection (VIP) scores. VIP > 1.0 are considered important predictors.

The Shapiro‒Wilk test confirmed deviations from normality (p < 0.05), justifying the use of Spearman’s correlation coefficient (r_s_). Moderately strong positive correlations were found between protein content and *Chl a* content (r_s_ = 0.57, p < 0.0001), as well as between protein content and *Chl a* + *b* content (r_s_ = 0.59, p < 0.0001). The correlation between protein content and *Chl b* content was weaker (r_s_ = 0.31, p < 0.0001), which is consistent with the known lower direct involvement of *Chl b* in photochemical reactions. The correlation between protein content and *Carot* content was weak and statistically nonsignificant (r_s_ = − 0.09, p > 0.05). Similarly, no significant relationship was found between protein content and the *Chl a/b* ratio (r_s_ = 0.07, p > 0.05). The PLS regression method was applied to comprehensively assess the influence of pigment traits on protein content of *H. helix* leaves and to identify the main predictors (Fig. 11a). The analysis of standardized regression coefficients in the PLS model indicated that the strongest positive predictors of protein content were the *Chl a* and *Chl a* + *b* contents (Fig. 11a). The Carot content had a pronounced negative effect on protein content, whereas the *Chl a/b* and *Chl b* contents had minimal effects. In the PLS model, the first latent component explained 42% of the variation among predictor variables (X) and 39% of the variation in the protein content (Y). The second component accounted for an additional 24% of the variation in X and 7% in Y. The greatest contribution to the prediction of protein content was made by the *Chl a* (VIP≈1.45) and *Chl a* + *b* (VIP≈1.45) contents (Fig. 11b). Other variables, including the *Chl b* content*,* Carot content, and *Chl a/b* ratio*,* had VIP values less than 0.8 and were considered minor predictors within this model.

### Relationships between SLA and protein content in *H. helix* leaves

Spearman’s correlation analysis revealed a weak positive correlation between SLA and protein content (r_s_ = 0.22, p < 0.01). The coefficient of determination was low (R^2^ = 0.016), indicating that SLA explains only a small portion of the variability in protein content (Fig. S3). The graphical analysis revealed that most observations were concentrated within the SLA range of 120–180 cm^2^ × g^−1^, where a moderate positive trend was observed, whereas at the extremes of SLA values, the protein content variability increased substantially without a clear pattern.

### Integrative response across biological scales

**Fig. 12:**
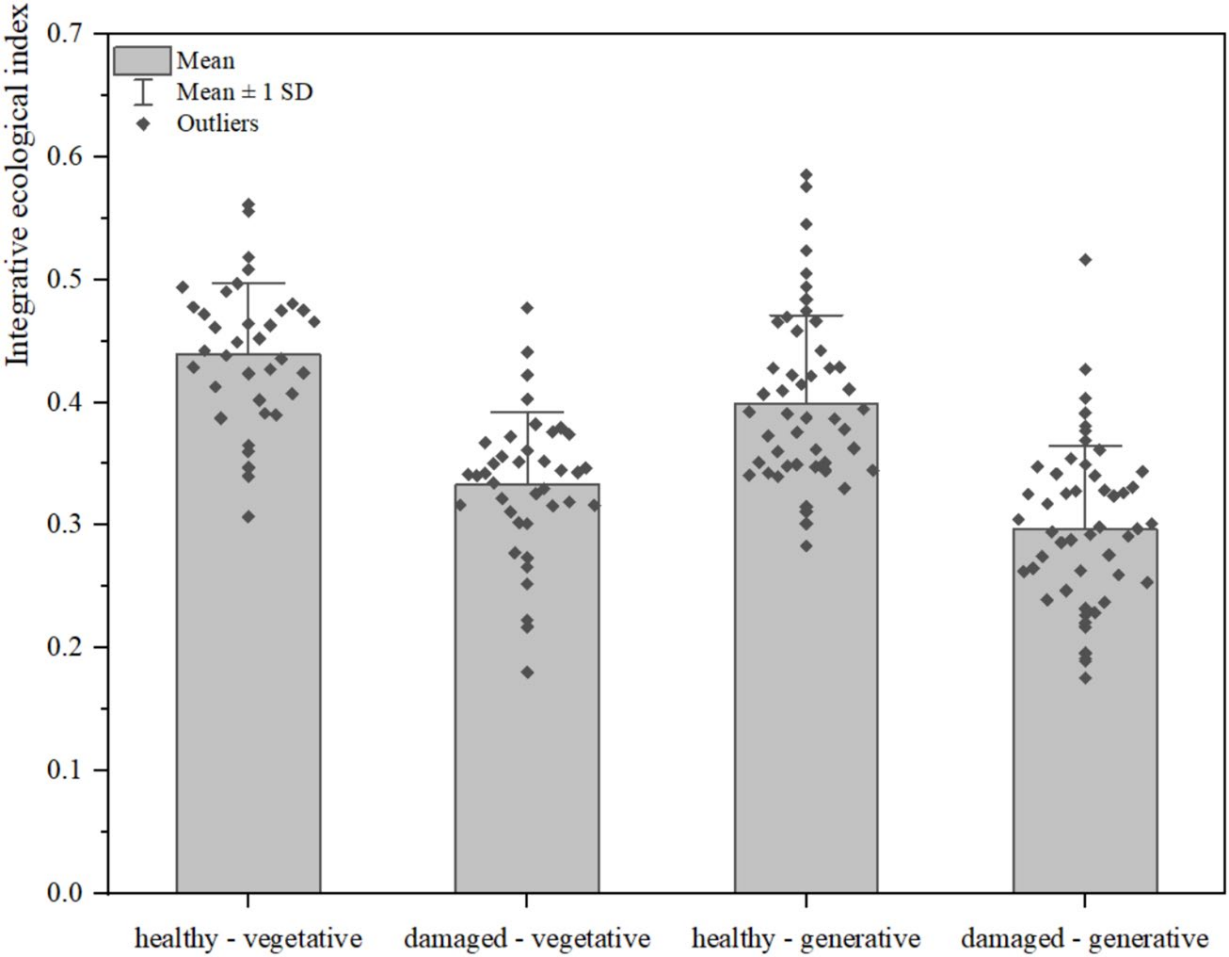
Integrative ecological index in *H. helix* leaves according to shoot type and leaf health.

The analysis of the integrative ecological index values using ANOVA and Tukey’s HSD revealed differences between *H. helix* groups (shoot type × leaf health, p < 0.0001) (Fig. 12). The highest mean index value was recorded for healthy leaves of vegetative shoots. The mean index value for the healthy leaves of generative shoots was slightly lower but remained comparable to that of vegetative shoots. The lowest mean index value was recorded for the damaged leaves of generative shoots. The greatest variability in the integrative ecological index, as indicated by the standard deviation and the distribution of outliers, was observed in both the healthy and damaged leaves of generative shoots.

Overall, these results highlight the complex morpho-functional organization of *H. helix* shoots in response to environmental gradients.

## Discussion

### Bioindicative potential of *H. helix* in temperate urban ecosystems

The adaptive responses of expansive species under urban conditions in temperate climates, particularly those of *H. helix*, remain insufficiently studied in relation to environmental factors. Given the existing evidence on the morphophysiological and biochemical responses of lianas to changing ecological conditions^11,40,45,63,79,80^, a multifunctional analysis of the responses of healthy and damaged leaves from vegetative and generative shoots remains a matter of ongoing discussion. The most pressing research need concerns the study of subpopulations of expansive lianas in urban environments, where in addition to abiotic drivers such as soil moisture, light availability, and temperature, biotic factors (e.g., competition, herbivory, and pathogens) and intense anthropogenic pressure^81^ also play substantial roles. One of the key challenges in ecological indication remains the selection of a system of interrelated responses from an indicator species that can reliably reflect changes in abiotic environmental factors^60^. Analyzing adaptive responses at a single level of biological organization does not allow for a comprehensive evaluation of the full spectrum of responses exhibited by the indicator. Modern functional ecology applies a methodological approach that integrates data from morphometry, anatomy, physiology, biochemistry, geobotany, and related disciplines^54,64,82^. An increase in the atmospheric CO₂ concentration has been shown to promote the spread and increase the density of lianas in both forest and urban environments by nearly 6.0% under temperate climate conditions^83^. Given this context, the adaptive response system of the expansive species *H. helix* may serve as an ecological indicator of environmental change in urban environments^21,84^.

### Functional differentiation and trait plasticity in *H. helix* subpopulations within urban ecosystems

Our analysis of the subpopulation composition of *H. helix* within natural clusters under urban conditions revealed diverse relationships between limiting and nonlimiting environmental factors (VWC, t, ES, and DLI) and the responses of morphotypes in ivy. For example, we observed a decrease in leaf density on generative shoots at heights up to 3 m, as well as a reduced proportion of damaged leaves at shaded or semishaded loci. Such spatial distribution patterns of leaves on generative and vegetative shoots in *H. helix* as a resource-conserving strategy are characteristic examples of ecological adaptation in many liana species^8,11,13,37,42,63,85,86^. In this study, the highest level of ecological variability was recorded for the leaf blade length (Ll), whereas the petiole length (Lp) was the most stable trait across both shoot types. The observed differences in the ecological plasticity indices of the leaf morphometric traits, in our view, indicate high morphological variability. This statement is supported by previously published data^87^. The morphometric response of liana leaves is supported by anatomical and physiological features of the leaf structure, such as the cuticle thickness and stomatal density^88^. Notably, attachment to physical supports induces the formation of lianescent xylem in lianas, which results in increased vessel diameter, broader vessel distribution, reduced fiber content, and higher potential specific hydraulic conductivity than the nonlianescent xylem in vegetative shoot types^89–91^. This pattern has also been confirmed for other expansive evergreen species^92,93^. These results indicate differential trait stability in response to environmental variation and provide valuable insight into the functional significance of morphometric adaptations in *H. helix* subpopulations within the ecosystems studied. Our analysis also revealed relationships between the biometric traits of *H. helix* shoots and environmental parameters. Strong correlations were recorded between soil temperature (t) and both the fresh and dry leaf biomasses of vegetative shoots. The daily light integral (DLI) was moderately negatively correlated with the fresh mass of the leaves and petioles of generative shoots. These findings are consistent with previously published data, which indicate that liana leaf biomass tends to decline under highly light-intensive conditions, elevated soil temperature, and reduced soil moisture content^11,42,94,95^. Other studies have demonstrated that in young liana individuals, increased light availability may promote biomass accumulation through resource reallocation to root systems, resulting in decreased LAR and SLA^96^. Under abiotic stress, biomass allocation between vegetative and generative shoots in lianas is a highly complex process that depends on the ontogenetic stage, ecological conditions, plant age, anthropogenic pressure, and other factors^45,81,87,89,90,97,98^. Compared with vegetative shoots, generative shoots are more tolerant to abiotic stress^11^. The variability observed in the morphometric and biometric traits of healthy and damaged leaves from both shoot types of *H. helix* in this study confirms their ecological plasticity within the range of environmental conditions found in urban park habitats.

### Hydration adjustment and functional plasticity under soil moisture gradients

Drought stress, particularly limited soil moisture, affects plant traits by reducing the stem length, total biomass, leaf area and leaf biomass and by disrupting the physiological mechanisms of water transport^99–104^. Water stress also impairs gas exchange processes, which leads to reduced growth and biomass accumulation. However, the growth of individuals of some species may increase under moderate soil moisture deficits^100^. The relative water content in plant organs is considered a reliable indicator of plant water status. Changes in ABWC, STWC, and LWC represent adaptive responses to water stress and are often used to identify plant genotypes with high drought tolerance^103^. Phenotypic plasticity is one of the key mechanisms through which plants respond to variations in their internal water content^73^. Studies have shown that when soil moisture decreases below optimal levels for a species, plants often reallocate resources (organic carbon) toward the root system. As a result, root biomass increases, whereas stem and leaf traits tend to decline^95,105–107^.

The results obtained in this study revealed relationships between the soil parameters (VWC, ES, and t) and functional traits of vegetative/generative shoots of ivy. VWC emerged as the key factor showing the strongest correlations with the relative water content in plant organs. Moderate positive correlations between VWC and LWC were found for both the healthy (r_s_ = 0.21) and damaged (r_s_ = 0.25) leaves of vegetative shoots. A similar trend was observed between VWC and ABWC, with the highest correlation (r_s_ = 0.48) recorded for vegetative shoots. Our findings are consistent with studies that show the high adaptive sensitivity of vegetative organs to variations in soil moisture^100,108^. Perennial lianas exhibit specific mechanisms for regulating transpiration and the water balance in response to soil moisture variation^109^. Under unfavorable drought conditions, osmotic regulation of the tissues of perennial lianas is increased^9,110,111^. The spatial distribution of liana loci explains more of the variation in leaf drought tolerance than phylogenetic relatedness does, suggesting that leaf drought tolerance is a highly variable trait^112^. The relationship between relative water content in generative shoot organs and VWC was weak. This result can be attributed to the anatomical and physiological characteristics of generative shoots, which are evolutionarily less dependent on environmental variations in favor of maintaining reproductive function^82^. We detected correlations between STWC/ABWC and t/ES for generative/vegetative shoots. These results may indicate that changes in osmotic mechanisms (increased osmotic activity in tissues to maintain the water balance in the presence of increasing ion concentrations in the soil) occur in both shoot types with minor variations in t/ES. Our assumption is supported by previous studies^113^.

### Functional trait coordination and resource-use trade-offs along abiotic gradients

A separate consideration of the relationships between soil moisture and functional traits of *H. helix* leaves is necessary. The present study confirms previous findings reported by our team^21,84^, demonstrating the dominant role of soil moisture as a limiting abiotic factor. Here, we confirmed the relationships between VWC and SLA/LMA. A moderate positive correlation between SLA and VWC and a corresponding moderate negative correlation between LMA and VWC indicate functional adaptation, i.e., the development of thinner leaves with larger surface areas. This result is consistent with the concept of resource-efficient plant strategies, where relatively high SLA values are typical under moist soil conditions^54,85,114,115^. The relationship between LMF and VWC suggests increased allocation to leaf biomass with relatively high soil moisture contents. As a result, photosynthetic function is enhanced, and energy/resources are redistributed toward expanding the assimilative surface^116^. These adaptations represent a typical expression of ecological plasticity in shade-tolerant species, such as *H. helix*. Moreover, these findings suggest that mineral availability may act as an additional limiting factor for *H. helix* subpopulation growth—an aspect that has been largely underrepresented in previous studies of this species under comparable ecological conditions. We concur that analyzing combinations of SLA, LMA, LMF, and LA allows the detection of structural–functional responses to soil moisture variations, thereby contributing to our understanding of species-level plasticity and its ecosystem-level implications^117^.

As part of this study, we showed that t and ES, which are nonlimiting environmental factors for the growth and development of ivy, do affect functional leaf traits. According to our results, the vector of LA was directed in the same orientation as those of ES and t, whereas the vector of SLA pointed in the opposite direction. This result indicates an ecological trade-off between maximizing the assimilative surface area (high SLA) and resource investment in leaf structural tissues^114,116,118,119^. A decrease in SLA and an increase in LA with increasing ES values reflect a shift from a rapid resource acquisition strategy toward a resource conservation strategy^85,116,119,120^. This conclusion aligns with studies addressing SLA–LMA changes in response to altered abiotic conditions^121^. Anatomical adjustments must also be considered, including reductions in leaf size and angle, stomatal positioning, epidermal thickness, and cuticle deposition^79,120^.

We found that the damaged leaves of generative shoots tended to present relatively high LMA values. This finding is consistent with studies reporting increased LMA and mesophyll thickness as adaptive responses to abiotic stress^114,121^. Similar ecological patterns have also been identified in other studies, which, for example, revealed a significant increase in LA in the presence of an elevated t^122^. From the perspective of functional specialization, this study revealed that the vegetative shoots of ivy are more sensitive to nonlimiting environmental factors. The generative shoots of ivy were relatively more stable and were less dependent on external conditions. The maintenance of homeostasis to preserve reproductive function under abiotic stress is widely recognized^11^. Overall, the adaptive responses observed in *H. helix* are not unique. Similar responses have been observed in vines of the genera *Parthenocissus* Planch. and *Echinocystis* Torr. & A. Grey^123^, as well as in tree species of the genera *Ailanthus* Desf*.*, *Phytolacca* L., and *Robinia* L., which exhibit changes in SLA, LMA, and LA in response to a range of abiotic stressors^124^.

### Shoot-type-specific photophysiological responses: pigment plasticity and hydration linkages

The evaluation of physiological traits is important for understanding how plants develop adaptive strategies under abiotic stress^101^. A low level of abiotic stress is essential for the normal functioning, growth, and development of plants^125–127^. One of the key physiological responses to abiotic stress is the alteration of chlorophyll content^128–132^. Chlorophylls are the most widespread plant pigments on Earth^133^. N. Smirnoff^134^ emphasized that abiotic stress can lead to the formation of reactive oxygen species (ROS) in chloroplasts as a result of excess chlorophyll excitation under conditions of limited photosynthesis. Abiotic stress may lead to reductions in chlorophyll content and dysfunction of photosystem II (PSII)^128,135–137^. A recent study showed that plants have evolved photoprotective mechanisms such as nonphotochemical quenching (NPQ) and alternative electron acceptors. Plants generate ROS that cause irreversible damage to the photosynthetic apparatus through photoinhibition of PSII^128^.

Changes in the pigment content in *H. helix* leaves provide additional insights into the adaptation of this species to abiotic stress conditions. The contents of photosynthetic pigments in ivy leaves depends on seasonal factors, with pigment profiles displaying clear dynamics, reaching a maximum in early autumn and a minimum during the winter–spring period^138^. Previous studies have shown that, compared with those cultivated in greenhouse environments, individuals growing under open-soil conditions presented greater variability in pigment content in response to changes in abiotic factors such as temperature, light intensity, and soil moisture^138^. Seasonal fluctuations in air temperature and soil irradiance may induce the excessive accumulation of reactive oxygen species (ROS) in the *H. helix* leaf cells, resulting in oxidative stress. This type of stress is characterized by increased membrane permeability and alterations in pigment content^139^. For example, elevated levels of malondialdehyde and hydrogen peroxide were observed during the winter period, alongside the activation of all major components of the antioxidant defense system, including superoxide dismutase, peroxidase, catalase, phenolic compounds, and carotenoids, as a protective physiological mechanism in ivy^139^. Researchers reported distinct physiological responses to changes in light intensity among different cultivars of *H. helix*^140^. The variegation patterns of the cultivars ‘Aureomarginata’ and ‘Golden Heart’ are determined by different mechanisms of morphogenesis and chloroplast differentiation. Under increasing light intensity, the function of the photosynthetic process (disruptions in chloroplast performance) changes; for example, paracrystalline structures are observed only in epidermal cells. This phenomenon is associated with light adaptation or photoprotective responses^140^. In addition, other studies have documented differences in the pigment content in *H. helix* leaves depending on the level of anthropogenic pressure^81^. In green areas outside urban zones, the chlorophyll content in generative shoots was higher than that in vegetative shoots. In contrast, under urban conditions, no significant differences in the chlorophyll content were detected between leaves from generative and vegetative shoots. The highest anthocyanin concentrations were recorded in the leaves of vegetative shoots collected from highly urbanized areas^81^. The results of this study confirmed the previously established relationship between DLI and pigment content in *H. helix* leaves. Reductions in Chl a, Chl b, and Chl a + b contents with high DLI values may indicate either pigment photodestruction or the disruption of chlorophyll biosynthesis. A decrease in pigment concentrations was observed in ivy leaves when DLI was ˃15 mol × m⁻^2^ × d⁻^1^. Up to a DLI of approximately 15 mol × m⁻^2^ × d⁻^1^, the pigment contents remained relatively stable, regardless of the shoot type. This trend is consistent with results suggesting the activation of photoprotective mechanisms (as evolutionary adaptations) in shade-tolerant lianas^86,98^.

A recent study demonstrated that *H. helix* exhibits high functional plasticity in response to extreme light conditions^141^. Under deep shade, the contents of Chl a and Chl b in leaves were 4.5 times higher than those in leaves under a high light intensity. This finding aligns with classical observations showing that shade-grown plants exhibit specific chloroplast adaptations—a dominance of photosystem II and elevated Chl a/b ratios. These adaptations allow them to maintain efficient photosynthetic activity under minimal photon flux^142^. An increased concentration of Chl b relative to Chl a may indicate ivy adaptation to high light availability, whereas lower Carot levels may suggest a reduced need for photoprotective mechanisms under ecologically optimal conditions for growth and development^138^. Shade-adapted species respond to low-light conditions by increasing the Chl b content and restructuring chloroplasts to increase the light absorption efficiency^143^. The efficiency of light intensity use in plants depends not only on chlorophyll content but also on the architecture of energy connectivity between photosystems^144^. One of the best-established markers of light adaptation is the increased abundance of the PsbS protein, a component involved in pH sensing within the thylakoid lumen, which triggers structural changes in the antenna complexes of photosystem II, ultimately leading to the dissipation of excess energy^145^. Considering the ecological plasticity of *H. helix* in response to light intensity, we hypothesized that a fast, PsbS-dependent energy quenching mechanism predominates in the leaves of generative shoots, whereas a slower, xanthophyll-dependent mechanism is more active in the leaves of vegetative shoots growing in shaded environments. This interpretation is consistent with the hypothesis of NPQ-related light plasticity in lianas, including *H. helix*, and supports the conclusions of Ramakers et al.^146^ regarding the differentiation of nonphotochemical quenching mechanisms depending on light intensity. Thus, the results of this study confirm the existence of complex physiological responses of *H. helix* to changing light intensity. The relationships between pigment contents and VWC should be considered. The Chl b content in *H. helix* leaves was particularly responsive to VWC. According to published data, drought stress leads to leaf yellowing, reduced activity of photosystem II (PSII), and a disruption of photosynthetic processes^147^. Other studies have shown that Chl a content may instead increase in response to moderate water deficit. This increase in Chl a content reflects an adaptive response aimed at maintaining photosynthetic activity under limited water availability^148^. In the present study, we identified relationships between LWC, STWC, ABWC and pigment contents in the healthy leaves of vegetative shoots. However, these correlations were weaker in the damaged leaves of generative shoots. A decrease in LWC corresponded with reductions in Chl a, Chl b, and Chl a + b contents^149^. The results of this study partially support our second hypothesis: while the coordination between LWC and pigment content was most pronounced in healthy vegetative leaves, certain tendencies were also observed in the leaves of generative shoots. Thus, this type of analysis warrants further study in future research. The established relationships highlight the importance of an integrative analysis of morphofunctional/physiological traits of leaves of vegetative/generative shoots to understand the adaptive mechanisms of lianas to abiotic stress under urban conditions. We supplemented the results of our previous studies^21,84^; the responses identified in this study represent not only intraspecific adaptations but also evolutionarily stable ecological strategies in plants^150^.

### Stress-related protein responses and multilevel integration of functional traits

The variations in protein contents among different shoot types and leaf conditions in *H. helix* provide important insights into the adaptive responses of this species under heterogeneous microhabitat conditions. The significantly higher protein concentrations observed in damaged generative leaves (mean, 248.14 mg × g⁻^1^ FW) than in the other groups likely reflect the activation of stress response pathways and intensified metabolic compensation mechanisms following tissue injury. These findings are consistent with the broader plant physiological patterns under abiotic stress described by Thapa and Shrestha^62^, who reported that stress conditions induce major modifications in protein metabolism, including increased synthesis of stress-responsive proteins, such as heat shock proteins (HSPs) and antioxidant enzymes. These proteins act to prevent aggregation, assist in proper folding, and help maintain photosynthetic efficiency under unfavorable conditions. Although *H. helix* was not experimentally exposed to controlled abiotic stress, elevated protein levels in mechanically damaged generative leaves may reflect similar defense-related protein metabolism processes. In contrast, healthy vegetative leaves presented the lowest mean protein content (210.08 mg × g⁻^1^ FW) and lowest variability, suggesting greater physiological stability and possibly lower baseline stress signaling. The absence of significant correlations between the protein content and abiotic drivers (DLI, VWC, t, and ES) suggests that short-term fluctuations in environmental conditions in urban park settings may not directly affect protein accumulation. Significant positive correlations between the protein content and pigment levels, particularly those of Chl a (r_s_ = 0.57) and Chl a + b (r_s_ = 0.59), indicate a close association between the photosynthetic apparatus and protein metabolism. This relationship supports previous findings by Tcherkez et al.^151^, who observed that increased photosynthetic activity was associated with increased translation and protein biosynthesis. The negligible influences of the carotenoid content and Chl a/b ratio on the variability in protein content likely reflects their roles in photoprotection rather than metabolic flexibility. Moreover, the weak but significant correlation between SLA and protein content (r_s_ = 0.22, p < 0.01) suggests that leaves with higher SLA values—and thus thinner structures—may exhibit slightly higher metabolic activity, although this relationship remains influenced by other variables, such as leaf age or local microdisturbances.

Integrated ecological assessment based on the normalization of several ecological indicators provides a multidimensional result^77^. The application of integrated ecological indices calculated from normalized environmental parameters such as vegetation indices, soil cover conditions, soil temperature and moisture, and anthropogenic pressure is an adaptive tool across diverse ecological conditions. For example, the enhanced ecological evaluation index has been proposed to assess ecosystem quality in different landscapes^78^. In modern analyses of abiotic stress impacts, a key priority is the integration of bioinformatics and artificial intelligence tools to enable a synergistic approach to the evaluation of sensitive morphological, functional, and genetic plant traits^152^. A recently proposed tool, the iPASTIC index calculator (a web-based application built using JavaScript and R), allows the calculation of plant tolerance indices to abiotic stress based on integrated indices^153^. The application of such indices is essential for studying the adaptations of expansive individuals across different levels of biological organization. A comprehensive assessment of factors influencing the functioning of lianas, such as soil conditions, topography, forest stand structure, and light intensity, is necessary to understand the expansive nature of lianas under varying environmental conditions^154^. According to the results of this study, the integrative ecological index based on z-normalization of the studied parameters effectively revealed the adaptive responses of the healthy and damaged leaves of vegetative/generative shoot types of *H. helix* to the environmental factors.

The traits of aboveground organs in lianas are not independent of one another, and their interrelationships vary depending on environmental conditions. The differentiation and integration of multiple liana traits under contrasting soil moisture conditions remain insufficiently studied^98^. Trait correlation variability tends to increase with the intensity of abiotic stressors. Such variability in trait relationships may help explain the expansive nature of many liana species^98^. Our results are in agreement with these conclusions. We found that under stress conditions (e.g., reduced soil moisture or a high soil light intensity), correlations among morphological, functional, and physiological traits were strengthened, particularly in the healthy leaves of vegetative shoots. From our point of view, trait differentiation and integration should be interpreted not only as a physiological response but also as an adaptive strategy of lianas in dynamic urban conditions.

Future research should explore whether the functional integration of traits also represents an adaptive mechanism in other urban lianas, such as *Parthenocissus* spp.

## Conclusions

This study provides a comprehensive, multilevel assessment of the ecophysiological plasticity of *H. helix* in response to heterogeneous environmental conditions in urban park ecosystems. By integrating morphometric, biochemical, pigmentary, and functional leaf traits alongside abiotic soil parameters and light availability, we demonstrated that both shoot type and leaf condition significantly influence the functional status of this expansive liana.

Key findings highlight the pivotal roles of leaf hydration and pigment composition in maintaining photosynthetic efficiency and structural integrity, especially under suboptimal conditions. Healthy vegetative shoots consistently presented stable trait profiles, whereas damaged generative shoots presented elevated variability and pronounced physiological adjustments, particularly in terms of the protein content, indicating intensified compensatory mechanisms under mechanical or environmental stress.

Despite the observed phenotypic differences, short-term abiotic fluctuations had limited direct effects on most functional traits. Instead, intrinsic plant factors and the shoot architecture appeared to be the primary drivers of adaptive responses. Notably, the integrative ecological index (IEI), developed from normalized trait-based subindices, effectively captured the multidimensional nature of adaptation and may serve as a promising tool for future ecological monitoring.

Taken together, these findings enhance our understanding of trait-based strategies underpinning the ecological success of *H. helix* in dynamic urban environments. The approach and indicators applied here have valuable potential for assessing plant resilience and for guiding green infrastructure planning under climate change and increasing anthropogenic disturbance.

## Supplementary Information

Supplementary Figure S1.

Supplementary Figure S2.

Supplementary Figure S3.

Supplementary Table S1.

Supplementary Table S2.

Supplementary Table S3.

Supplementary Table S4.

Supplementary Table S5.

## Data Availability

The datasets used and/or analysed during the current study available from the corresponding author on reasonable request.
