# Supplementary material for: Multilevel trait responses of liana Hedera helix L. to environmental gradients in urban forest ecosystems
Source: Sci Rep. 2025 Nov 17;15:40155. doi: 10.1038/s41598-025-23815-0 (PMC12623917; doi:10.1038/s41598-025-23815-0)

Figure S1.

Ground and trunk cover by *Hedera helix* L. in urban forests, Poznań, Poland (study sites, June 2024)

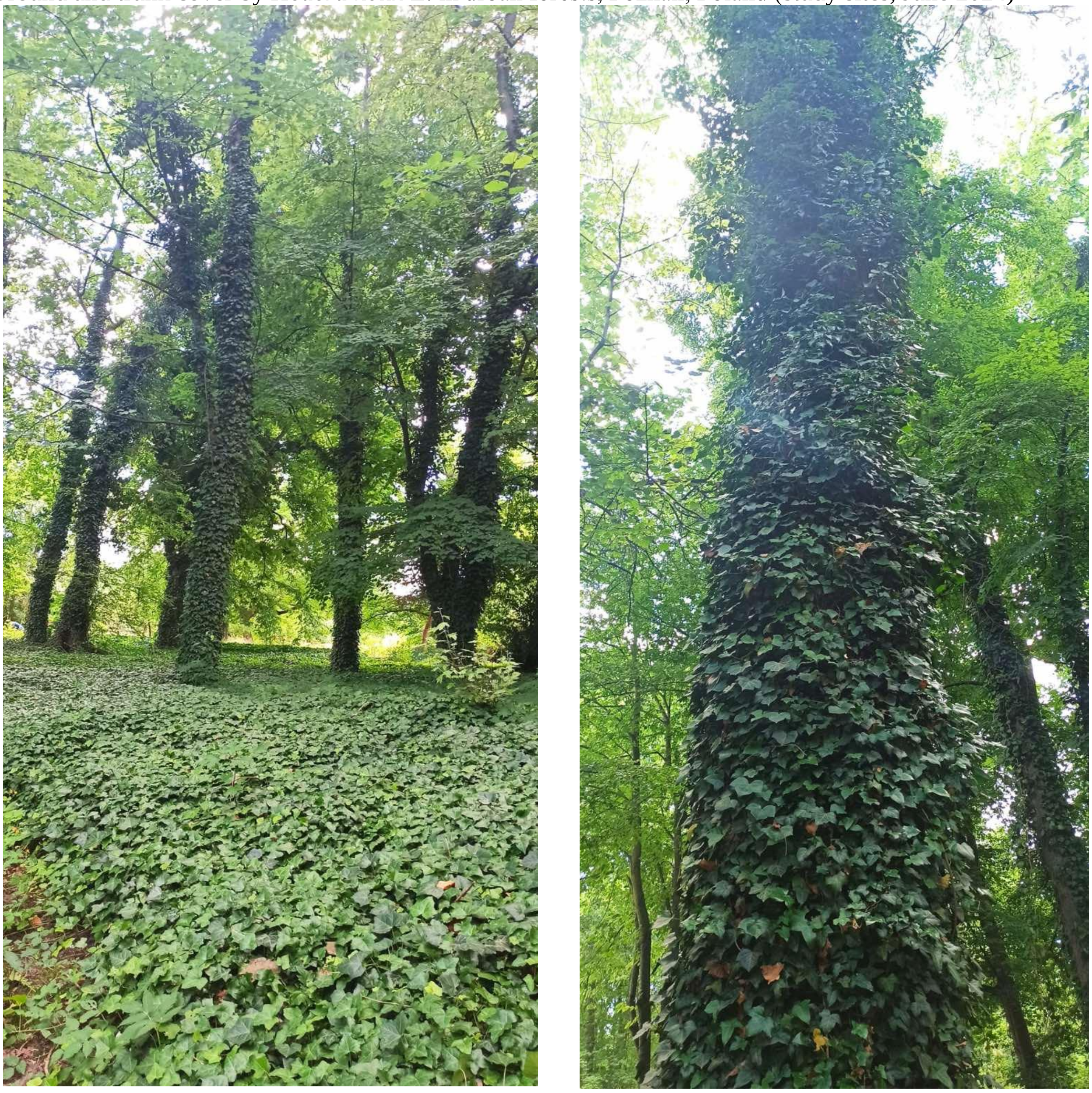

Supplement: Supplementary file 1 — Supplementary Figure S1. [file 41598_2025_23815_MOESM1_ESM.pdf]
