# Supplementary material for: Multilevel trait responses of liana Hedera helix L. to environmental gradients in urban forest ecosystems
Source: Sci Rep. 2025 Nov 17;15:40155. doi: 10.1038/s41598-025-23815-0 (PMC12623917; doi:10.1038/s41598-025-23815-0)
Supplement: Supplementary file 2 — Supplementary Figure S2. [file 41598_2025_23815_MOESM2_ESM.pdf]

Figure S2.

Variance explained by canonical axes in canonical correlation analysis (CCA). **(a)** Proportion and cumulative variance explained by the canonical components for environmental predictor variables (t, EC, VWC, DLI); **(b)** Proportion and cumulative variance explained for pigment response variables (*Chl a*, *Chl b*, *Chl a + b*, *Carot*, and *Chl a/b*).

Note: Figure presents total explained variance per canonical axis, not per individual pigment trait

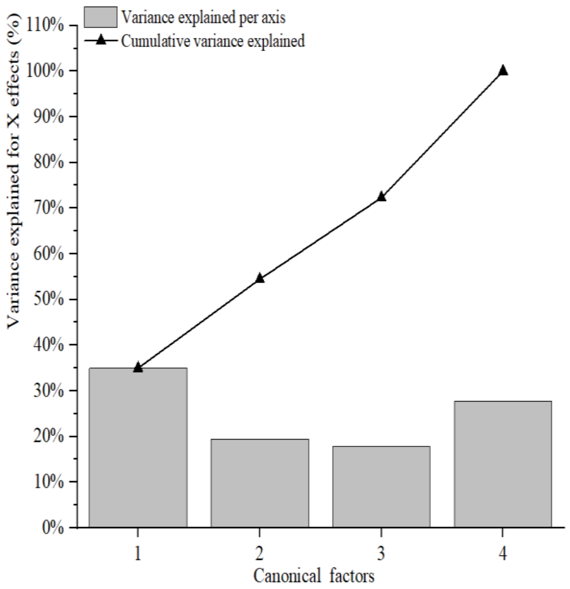

a

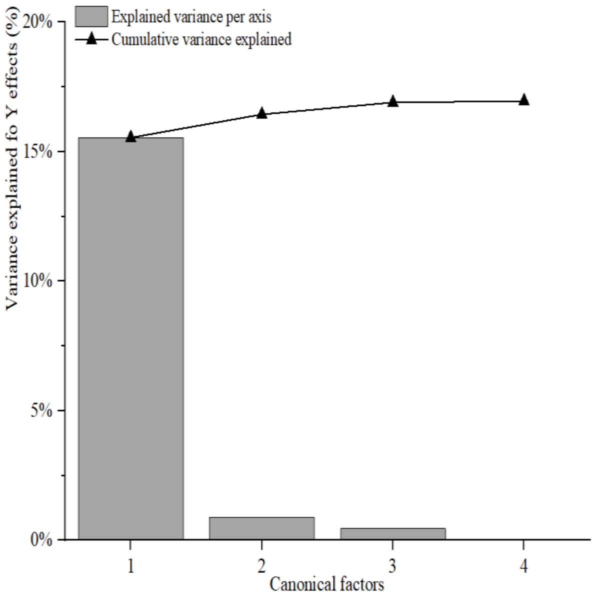

b
