# Supplementary figures and images for: Multilevel trait responses of liana Hedera helix L. to environmental gradients in urban forest ecosystems
Source: Sci Rep. 2025 Nov 17;15:40155. doi: 10.1038/s41598-025-23815-0 (PMC12623917; doi:10.1038/s41598-025-23815-0)

**Figure S3.**

Relationship between protein content in *H. helix* leaves and SLA

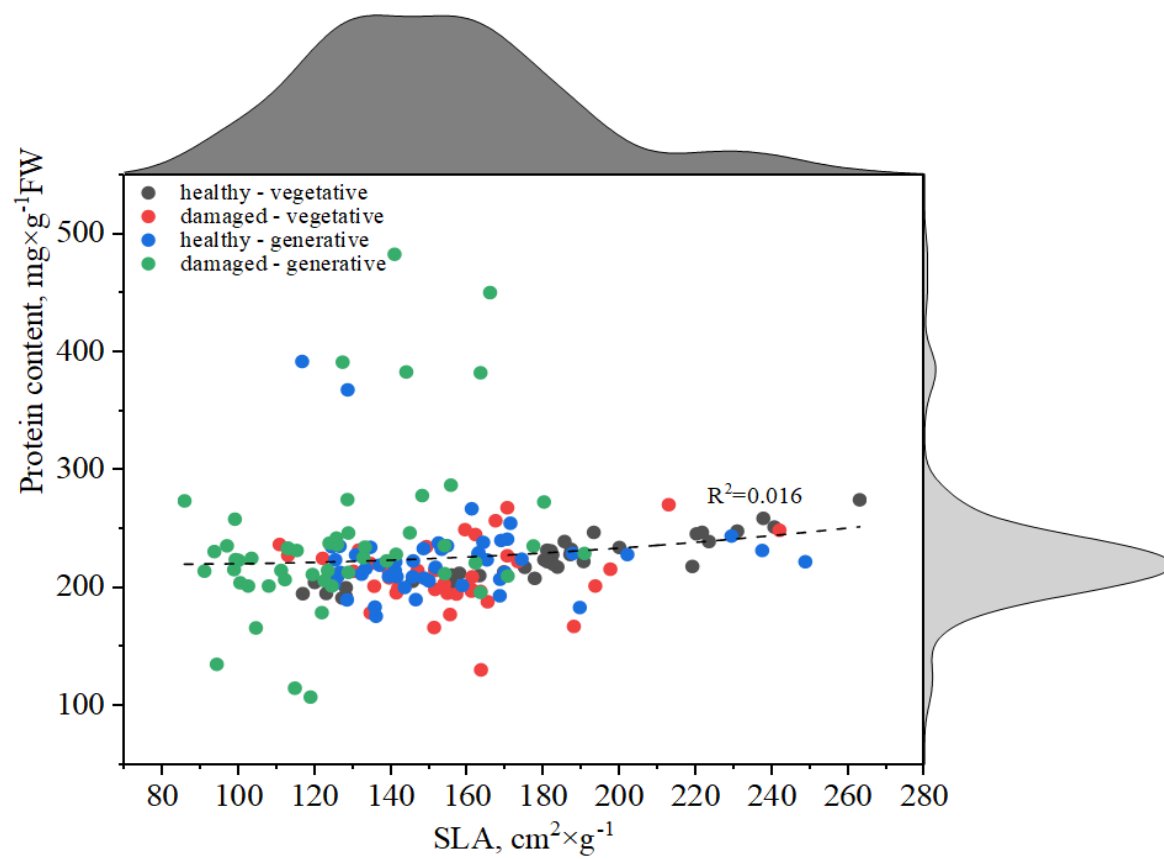

Supplement: Supplementary file 3 — Supplementary Figure S3. [file 41598_2025_23815_MOESM3_ESM.pdf]
