# Supplementary material for: Multilevel trait responses of liana Hedera helix L. to environmental gradients in urban forest ecosystems
Source: Sci Rep. 2025 Nov 17;15:40155. doi: 10.1038/s41598-025-23815-0 (PMC12623917; doi:10.1038/s41598-025-23815-0)
Supplement: Supplementary file 4 — Supplementary Table S1. [file 41598_2025_23815_MOESM4_ESM.docx]

**Table S1.**

Definitions, abbreviations, and units for the studied traits of individual *H. helix*

| **Trait** | **Abbr.** | **Definition** | **Unit** |
| --- | --- | --- | --- |
| Leaf area | LA | Leaf surface area | cm^2^ |
| Leaf mass per area | LMA | The leaf dry biomass per unit leaf area | g×cm^-2^ |
| Leaf mass fraction | LMF | The ratio of the leaf dry biomass to the dry aboveground biomass of an individual | g×g^-1^ |
| Specific leaf area | SLA | The ratio of the leaf area to the leaf dry biomass | cm^2^×g |
| Leaf mass | L_m_ | Total mass of leaves collected from SP | g |
| Stem mass | S_m_ | Total mass of stems collected from SP | g |
| Leaf petiole mass | L_pm_ | Total mass of leaf petioles from SP | g |
| Leaf petiole length | L_p_ | Length of the leaf petiole from the sampled shoot | cm |
| Leaf length | L_l_ | Length of the leaf blade from the sampled shoot | cm |
| Leaf width | W_l_ | Width of the leaf blade from the sampled shoot | cm |
