# Supplementary material for: Multilevel trait responses of liana Hedera helix L. to environmental gradients in urban forest ecosystems
Source: Sci Rep. 2025 Nov 17;15:40155. doi: 10.1038/s41598-025-23815-0 (PMC12623917; doi:10.1038/s41598-025-23815-0)
Supplement: Supplementary file 5 — Supplementary Table S2. [file 41598_2025_23815_MOESM5_ESM.docx]

**Table S2.**

Formulas and lists of traits used to calculate the analytical subindices of the Integrative Ecological Index (IEI)

| **Subindex** | **Abbr.** | **Formula** | **Traits included** |
| --- | --- | --- | --- |
| Water stress index | WI | $WI=\frac{Z_{\mathrm{LWC}}+Z_{\mathrm{STWC}}+Z_{\mathrm{ABWC}}}{3}$ | Leaf water content (LWC), Stem water content (STWC), Aboveground biomass water content (ABWC) |
| Functional leaf trait index | FI | $FI=\frac{Z_{\mathrm{SLA}}+Z_{\mathrm{LMA}}+Z_{\mathrm{LMF}}}{3}$ | Specific leaf area (SLA), Leaf mass per area (LMA), Leaf mass fraction (LMF) |
| Pigment index | PgI | $PgI=\frac{Z_{Chl a}+Z_{Chl b}+Z_{\mathrm{Carot}}}{3}$ | Chlorophyll a (Chl a), Chlorophyll b (Chl b), Сarotenoids (Carot) |
| Protein index | PrI | $PrI=Z_{\mathrm{PC}}$ | Protein content (PC) |
