# Supplementary material for: Multilevel trait responses of liana Hedera helix L. to environmental gradients in urban forest ecosystems
Source: Sci Rep. 2025 Nov 17;15:40155. doi: 10.1038/s41598-025-23815-0 (PMC12623917; doi:10.1038/s41598-025-23815-0)
Supplement: Supplementary file 6 — Supplementary Table S3. [file 41598_2025_23815_MOESM6_ESM.docx]

**Table S3.**

P values corresponding to Spearman’s rank correlation coefficients between environmental variables (t, ES, and VWC) and leaf hydration parameters (LWC, STWC, and ABWC) in *H. helix*.

| Pair | | Group | | | |
| --- | --- | --- | --- | --- | --- |
|  |  | healthy vegetative | damaged vegetative | healthy generative | damaged generative |
| t | LWC | p<0.001 | p<0.01 | p˃0.05 | p˃0.05 |
|  | STWC | p<0.05 | p<0.01 | p<0.01 | p<0.01 |
|  | ABWC | p˃0.05 | p<0.05 | p˃0.05 | p˃0.05 |
| VWC | LWC | p<0.01 | p<0.01 | p˃0.05 | p˃0.05 |
|  | STWC | p<0.05 | p˃0.05 | p<0.01 | p˃0.05 |
|  | ABWC | p<0.001 | p˃0.05 | p<0.01 | p˃0.05 |
| ES | LWC | p˃0.05 | p˃0.05 | p˃0.05 | p˃0.05 |
|  | STWC | p˃0.05 | p˃0.05 | p<0.05 | p<0.01 |
|  | ABWC | p<0.05 | p<0.05 | p<0.05 | p<0.05 |
