# Supplementary material for: Multilevel trait responses of liana Hedera helix L. to environmental gradients in urban forest ecosystems
Source: Sci Rep. 2025 Nov 17;15:40155. doi: 10.1038/s41598-025-23815-0 (PMC12623917; doi:10.1038/s41598-025-23815-0)
Supplement: Supplementary file 7 — Supplementary Table S4. [file 41598_2025_23815_MOESM7_ESM.docx]

**Table S4.**

Biometric and morphometric characteristics of H. helix shoots

| **Mass** | | **Health of leaves** | **Type**  **of shoot** | **Mean** | **SD** | **Coefficient of variation** | **Min** | **Med** | **Max** |
| --- | --- | --- | --- | --- | --- | --- | --- | --- | --- |
| L_m_ | fresh | healthy | vegetative | 102.62 | 24.55 | 0.23 | 43.32 | 98.11 | 170.33 |
|  |  |  | generative | 48.00 | 36.03 | 0.75 | 4.056 | 37.29 | 160.62 |
|  |  | damaged | vegetative | 12.21 | 6.04 | 0.49 | 5.16 | 10.76 | 35.87 |
|  |  |  | generative | 7.03 | 4.57 | 0.65 | 1.85 | 5.62 | 21.78 |
|  | dry | healthy | vegetative | 32.28 | 7.72 | 0.24 | 12.89 | 31.47 | 54.12 |
|  |  |  | generative | 17.46 | 13.46 | 0.77 | 1.38 | 12.28 | 60.00 |
|  |  | damaged | vegetative | 4.40 | 2.68 | 0.61 | 1.80 | 3.29 | 13.11 |
|  |  |  | generative | 2.72 | 2.05 | 0.75 | 0.45 | 1.98 | 9.67 |
| S_m_ | fresh | healthy | vegetative | 151.11 | 75.67 | 0.50 | 49.16 | 153.51 | 312.47 |
|  |  |  | generative | 58.63 | 49.39 | 0.84 | 7.61 | 31.88 | 189.37 |
|  |  | damaged | vegetative | 148.52 | 68.03 | 0.45 | 48.16 | 150.67 | 318.02 |
|  |  |  | generative | 57.18 | 49.05 | 0.86 | 7.61 | 31.17 | 189.37 |
|  | dry | healthy | vegetative | 45.25 | 23.08 | 0.51 | 14.81 | 48.32 | 108.50 |
|  |  |  | generative | 24.07 | 20.89 | 0.87 | 3.45 | 12.93 | 84.87 |
|  |  | damaged | vegetative | 49.68 | 25.74 | 0.52 | 13.61 | 48.37 | 108.50 |
|  |  |  | generative | 23.22 | 20.42 | 0.88 | 3.45 | 13.73 | 84.87 |
| L_рm_ | fresh | healthy | vegetative | 79.85 | 23.63 | 0.30 | 33.22 | 78.09 | 143.64 |
|  |  |  | generative | 8.22 | 6.50 | 0.79 | 0.49 | 6.23 | 25.76 |
|  |  | damaged | vegetative | 77.03 | 23.41 | 0.30 | 33.22 | 76.60 | 143.64 |
|  |  |  | generative | 8.19 | 5.63 | 0.68 | 0.49 | 7.05 | 25.76 |
|  | dry | healthy | vegetative | 15.17 | 4.37 | 0.29 | 6.05 | 14.65 | 25.19 |
|  |  |  | generative | 2.43 | 2.01 | 0.83 | 0.16 | 1.837 | 9.12 |
|  |  | damaged | vegetative | 14.56 | 4.24 | 0.29 | 7.05 | 14.56 | 24.77 |
|  |  |  | generative | 2.53 | 1.82 | 0.72 | 0.16 | 1.99 | 7.03 |
| L_p_ | fresh | healthy | vegetative | 10.33 | 4.02 | 0.39 | 5.78 | 9.71 | 13.51 |
|  |  |  | generative | 11.21 | 4.18 | 0.37 | 6.02 | 10.11 | 14.01 |
|  |  | damaged | vegetative | 10.46 | 3.24 | 0.31 | 5.56 | 9.37 | 12.44 |
|  |  |  | generative | 11.02 | 3.20 | 0.29 | 6.11 | 10.28 | 10.75 |
| L_l_ | fresh | healthy | vegetative | 6.43 | 0.81 | 0.13 | 4.23 | 5.77 | 7.25 |
|  |  |  | generative | 7.25 | 1.21 | 0.17 | 5.33 | 7.09 | 7.84 |
|  |  | damaged | vegetative | 5.48 | 0.96 | 0.18 | 4.28 | 5.15 | 6.74 |
|  |  |  | generative | 6.76 | 1.22 | 0.18 | 5.12 | 6.41 | 8.98 |
| W_l_ | fresh | healthy | vegetative | 6.02 | 1.34 | 0.22 | 3.02 | 5.56 | 8.23 |
|  |  |  | generative | 5.39 | 2.10 | 0.39 | 2.99 | 5.21 | 7.86 |
|  |  | damaged | vegetative | 5.74 | 1.60 | 0.28 | 3.01 | 5.38 | 6.75 |
|  |  |  | generative | 5.32 | 2.34 | 0.44 | 2.54 | 5.14 | 6.98 |

***Note:*** L_m_, leaf mass, g; S_m_, stem mass, g; L_pm_, leaf petiole mass, g; L_p_, petiole length, cm; L_l_, leaf blade length, cm; W_l,_ leaf blade width, cm.
