# Supplementary material for: Multilevel trait responses of liana Hedera helix L. to environmental gradients in urban forest ecosystems
Source: Sci Rep. 2025 Nov 17;15:40155. doi: 10.1038/s41598-025-23815-0 (PMC12623917; doi:10.1038/s41598-025-23815-0)
Supplement: Supplementary file 8 — Supplementary Table S5. [file 41598_2025_23815_MOESM8_ESM.docx]

**Table S5**

Results of the one-way ANOVA for water content parameters (LWC, STWC, and ABWC) in *H. helix* shoots

| **Parameter** | **Group** | **Mean (%)** | **SD** | **F** | **p value** |
| --- | --- | --- | --- | --- | --- |
| LWC | healthy – vegetative | 67.65 | 2.61 | 7.65 | <0.0001 |
|  | damaged – vegetative | 64.41 | 2.22 |  |  |
|  | healthy – generative | 64.25 | 1.89 |  |  |
|  | damaged – generative | 61.29 | 10.95 |  |  |
| STWC | healthy – vegetative | 73.09 | 2.94 | 100.27 | <0.0001 |
|  | damaged – vegetative | 77.07 | 2.69 |  |  |
|  | healthy – generative | 61.16 | 6.66 |  |  |
|  | damaged – generative | 61.39 | 6.65 |  |  |
| ABWC | healthy – vegetative | 69.34 | 1.99 | 29.31 | <0.0001 |
|  | damaged – vegetative | 66.94 | 5.44 |  |  |
|  | healthy – generative | 60.83 | 5.24 |  |  |
|  | damaged – generative | 57.99 | 9.49 |  |  |
